# Transcriptomic profiling of SARS-CoV-2 infected human cell lines identifies HSP90 as target for COVID-19 therapy

**DOI:** 10.1016/j.isci.2021.102151

**Published:** 2021-02-06

**Authors:** Emanuel Wyler, Kirstin Mösbauer, Vedran Franke, Asija Diag, Lina Theresa Gottula, Roberto Arsiè, Filippos Klironomos, David Koppstein, Katja Hönzke, Salah Ayoub, Christopher Buccitelli, Karen Hoffmann, Anja Richter, Ivano Legnini, Andranik Ivanov, Tommaso Mari, Simone Del Giudice, Jan Papies, Samantha Praktiknjo, Thomas F. Meyer, Marcel Alexander Müller, Daniela Niemeyer, Andreas Hocke, Matthias Selbach, Altuna Akalin, Nikolaus Rajewsky, Christian Drosten, Markus Landthaler

**Affiliations:** 1 Berlin Institute for Medical Systems Biology, Max-Delbrück-Center for Molecular Medicine in the Helmholtz Association, Hannoversche Str 28, 10115 Berlin, Germany; 2 Institute of Virology, Charité-Universitätsmedizin Berlin and Berlin Institute of Health, Charitéplatz 1, 10117 Berlin, Germany; 3 Max-Delbrück-Center for Molecular Medicine in the Helmholtz Association, Robert-Rössle-Strasse 10, 13125 Berlin, Germany; 4 Department of Pediatrics, Charité – University Hospital Berlin, 13353 Berlin, Germany; 5 Department of Internal Medicine/Infectious Diseases and Pulmonary Medicine, Charité University Medicine, Berlin, Germany; 6 Core Unit Bioinformatics, Berlin Institute of Health, Charité – University Hospital Berlin, 10117 Berlin, Germany; 7 Laboratory of Infection Oncology, Institute of Clinical Molecular Biology, UKSH, Christian Albrechts University of Kiel, 24105 Kiel, Germany; 8 IRI Life Sciences, Institut für Biologie, Humboldt Universität zu Berlin, Philippstraße 13, 10115 Berlin, Germany

**Keywords:** Biological Sciences, Virology, Omics, Transcriptomics

## Abstract

Detailed knowledge of the molecular biology of severe acute respiratory syndrome coronavirus 2 (SARS-CoV-2) infection is crucial for understanding of viral replication, host responses, and disease progression. Here, we report gene expression profiles of three SARS-CoV- and SARS-CoV-2-infected human cell lines. SARS-CoV-2 elicited an approximately two-fold higher stimulation of the innate immune response compared to SARS-CoV in the human epithelial cell line Calu-3, including induction of miRNA-155. Single-cell RNA sequencing of infected cells showed that genes induced by virus infections were broadly upregulated, whereas interferon beta/lambda genes, a pro-inflammatory cytokines such as IL-6, were expressed only in small subsets of infected cells. Temporal analysis suggested that transcriptional activities of interferon regulatory factors precede those of nuclear factor κB. Lastly, we identified heat shock protein 90 (HSP90) as a protein relevant for the infection. Inhibition of the HSP90 activity resulted in a reduction of viral replication and pro-inflammatory cytokine expression in primary human airway epithelial cells.

## Highlights

• Transcriptomic landscape of human cell lines infected with SARS-CoV or SARS-CoV-2
• Stronger innate immune response to SARS-CoV-2 and induction of miR-155
• Transcription of interferon gene is transient and followed by NF-kB target genes
• Hsp90 inhibitors impair viral replication and induction of pro-inflammatory genes

## Graphical abstract

**Figure:**
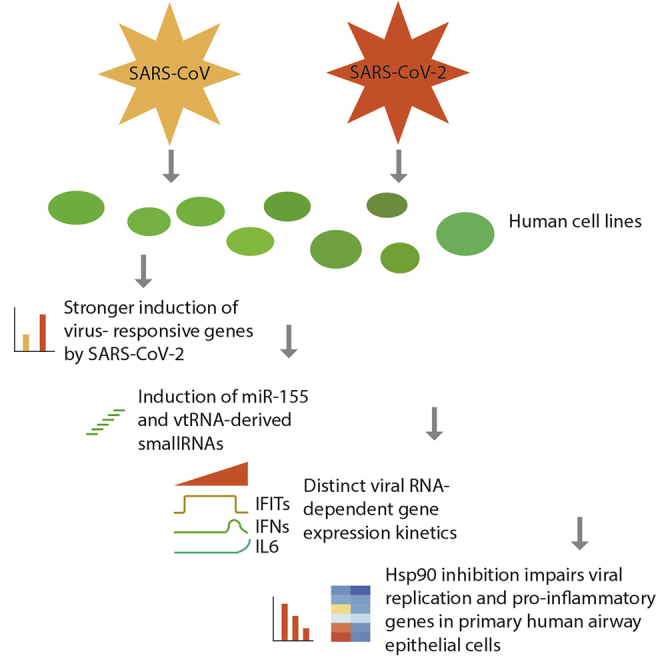

## Introduction

Diseases caused by coronaviruses (CoVs) range from asymptomatic and mild infections of the upper respiratory tract to severe acute respiratory distress, when the lower respiratory tract is infected. In addition to the six previously known CoVs affecting humans, a novel CoV termed severe acute respiratory syndrome coronavirus 2 (SARS-CoV-2) has recently emerged. The novel SARS-CoV-2, which causes coronavirus disease 2019 (COVID-19), is an ongoing global health threat since the beginning of the outbreak in late 2019 and has, at the time of writing this text, infected more than 80 million people worldwide (World Health Organization, 2020). The SARS-CoV-2 life cycle initiates with the attachment of the virion to the cell surface and subsequent binding to the angiotensin converting enzyme 2 (ACE2), followed by proteolytic cleavage and internalization (Fehr and Perlman, 2015; Hoffmann et al., 2020; Masters and Perlman, 2013). Non-structural proteins are then translated to form a replicase-transcriptase complex, in which the full genomic RNA, as well as subgenomic RNAs are generated within double membrane vesicles (DMVs) (Fehr and Perlman, 2015; Masters and Perlman, 2013; van Hemert et al., 2008). Incoming viral RNA is detected by sensors such as IFIH1 (interferon induced with helicase C domain 1; also known as MDA5) and DDX58 (DExD/H-Box helicase 58; also known as RIG-I), which trigger the antiviral response. This sensing and signaling is impaired by a range of viral factors, e.g. replication within DMVs, RNA capping, and methylation, or shortening of the poly-U tail on the minus strand RNA (Hackbart et al., 2020; Menachery et al., 2014a). Furthermore, inhibition of interferon regulatory factor (IRF) activity (Spiegel et al., 2005) and a delayed induction of interferon-stimulated genes (ISGs) compared to influenza virus infection or type I interferon treatment itself (Menachery et al., 2014b) was observed in SARS-CoV infection. Importantly, accessory genes in the SARS-CoV genome, like open reading frame 6 (ORF6), may code for antagonists of interferon signaling (Narayanan et al., 2008).

Following production of subgenomic RNAs, during which a constant 5′ leader is prepended by a process called discontinuous transcription (Sola et al., 2015), the viral genes are translated either in the cytoplasm, e.g. the nucleocapsid protein, N, or at the endoplasmic reticulum (ER), such as the envelope (E), membrane (M), and spike (S) proteins (Alsaadi and Jones, 2019; Muller et al., 2010). The substantial increase in ER translation causes ER stress, which triggers the unfolded protein response (UPR). This is then integrated with double-stranded RNA sensing at the level of eukaryotic initiation factor 2 alpha (eIF2alpha) phosphorylation (Fung and Liu, 2014). The ER stress response is likely attenuated by the viral E protein (DeDiego et al., 2011; Li et al., 2019). Accordingly, heat shock proteins (HSPs), which ameliorate ER stress, have been described to be generally relevant for virus infections (Santoro et al., 2010). Furthermore, ER stress induces autophagy, a cell recycling pathway which can be used by some viruses for productive replication (Guo et al., 2017). Finally, dysregulation of microRNA (miRNA) expression and subsequent alterations in gene expression patterns have also been reported to play a role in infected cells (Kemp et al., 2020; Leon-Icaza et al., 2019).

Comprehensive profiling of SARS-CoV-2-mediated perturbations of gene expression are at the beginning. A recent in-depth analysis of the transcriptional response to SARS-CoV-2 in comparison to other respiratory viruses in cells and animal models revealed a virus-specific inflammatory response (Blanco-Melo et al., 2020). For further in-depth studies, high-resolution methods such as single-cell RNA-sequencing (scRNA-seq) are of particular interest. They allow the characterization of cellular heterogeneity over the course of infection, which may be masked at the population level (Cristinelli and Ciuffi, 2018; Drayman et al., 2019; Russell et al., 2018; Shnayder et al., 2018; Wyler et al., 2019; Zanini et al., 2018). Furthermore, techniques such as small RNA sequencing, which reveals miRNAs and other small RNAs (Bartel, 2004; Friedlander et al., 2012), allow the characterization of other aspects influencing the regulation of gene expression.

Here, we performed a comprehensive analysis of three human cell lines infected with SARS-CoV or SARS-CoV-2, namely the gut cell line Caco-2, as well as the lung cell lines Calu-3 and H1299. We generated scRNA-seq, poly(A)^+^, and total RNA transcriptomic data, as well as small RNA profiling in infection time courses for both viruses.

Efficiency and productivity of infection as well as the interferon response was remarkably different between cell lines. Interestingly, SARS-CoV-2 induced a two-fold higher expression of genes induced by the infection than SARS-CoV. In addition, we found strong induction of miR-155 with both viruses, suggesting a role for this miRNA in the progression of infection. The scRNA-seq data showed that while some genes such as interferon-induced protein with tetratricopeptide repeats 1 and 2 (IFIT1/IFIT2) were broadly induced, interferon beta (IFNB1) and interleukin-6 (IL6) were expressed only in subsets of infected cells. Furthermore, the transcriptional induction of nuclear factor-κB (NF-κB) targets could be temporally separated from the interferon-driven transcription. Detailed investigations of cellular gene expression programs suggest an involvement of the protein folding chaperone and autophagy regulator HSP90 in the viral infection cycle. Inhibition of HSP90 by multiple inhibitors resulted in reduced viral replication and cytokine mRNA levels. Overall, our study provides a detailed picture of the gene expression changes in cell line models for CoVs and particularly SARS-CoV-2, highlights the cell-type specificity of the transcriptional response to infection and identifies HSP90 as a potential target for therapeutic interventions.

## Results

### Different permissiveness of SARS-CoV-2 infection in cell lines

To establish cell culture systems for studying SARS-CoV-2 replication and host cell responses, we examined the epithelial lung cancer cell lines, H1299 and Calu-3, since infection of lung epithelial cells is a key feature of SARS-CoV-2 infection in the human body. In addition, we used the epithelial colorectal adenocarcinoma cell line, Caco-2, which is frequently used as a CoV cell culture model (Bojkova et al., 2020; Chu et al., 2020; Klann et al., 2020; Reigel, 1985), and infection of intestinal cells is still under debate (Lamers et al., 2020). Transfection of poly-I:C RNA resulted in induction of IFIT1, IFIT2, and OAS2 (2′-5′-Oligoadenylate Synthetase 2) genes in Calu-3 and H1299 cells, indicating that sensing of foreign RNA in the cytosol is active in these cell types. This response was not observed in Caco-2 cells (Figure S1A), which exhibit low expression of viral RNA receptor genes, IFIH1/MDA5 and DDX58/RIG-I (Figure S2C).

**Figure 1:**
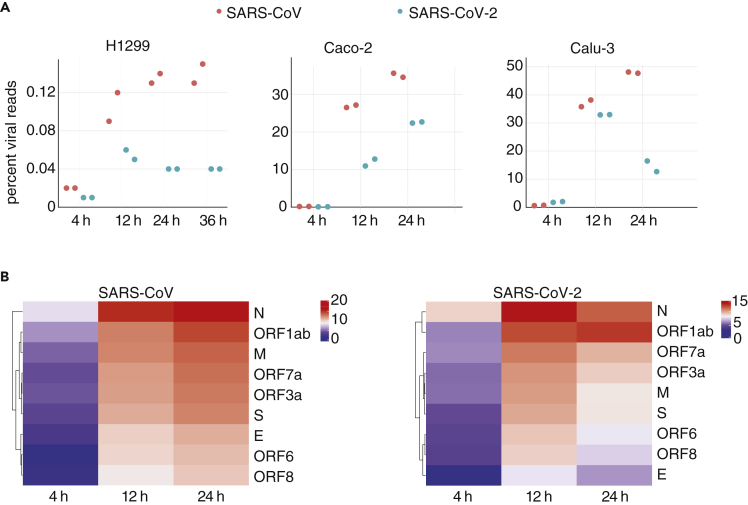
Different permissiveness of SARS-CoV/-2 infection in cell lines (A) Viral read percentages of total reads of the respective cell line at different time points of infection. Note that Calu-3 cells infected with SARS-CoV-2 show clear signs of cell death at 24 hpi (Figure S1I), which likely influences the viral yield at the latest time point. (B) Heatmaps of canonical junction-spanning viral reads in total RNA from Calu-3 cells infected with either virus, averaged across biological replicates per time point, expressed in log_2_(TMM-normalized counts per million).

For all cell lines, we performed a comprehensive analysis of transcriptome changes. Cells were infected with either SARS-CoV (Frankfurt strain) or SARS-CoV-2 (patient isolate BetaCoV/Munich/BavPat1/2020|EPI_ISL_406862) at an MOI of 0.33 (schematic representation of experiments in Figure S1B and Table S1), and sampled at different time points after infection. The percentages of viral transcripts in intracellular RNAs, as determined by poly(A)^+^ and RNA-seq sequencing were low in H1299 cells for both viruses, in contrast to Caco-2 and Calu-3 cells (Figure 1A, Table S2). Accordingly, the yield of infectious virus particles was higher for the permissive cell lines Caco-2 and Calu-3 (Figure S1C). The low susceptibility of H1299 cells might, at least partially, be attributed to the low expression of the SARS-CoV receptor ACE2, as suggested by the RNA-sequencing data and Western blot analysis (Figure S1D).

By counting poly(A)^+^ or total RNA-seq reads spanning the junction of the viral leader and its downstream gene (82.6% of virus-mapping split reads), we accurately quantified the relative amounts of subgenomic viral mRNAs (Irigoyen et al., 2016; Kim et al., 2020). We observed a consistent, time-dependent hierarchy of gene expression, mostly dominated by viral mRNAs encoding the N gene (Figures 1B and S1E–S1H), similar to a recent report for the alpha Human CoV-229E (HCoV-229E) (Viehweger et al., 2019). At later time points post infection, the relative amount of ORF7a generally increased. Notably, this approach failed to detect expression of leaders immediately adjoining ORF7b or ORF10 (Table S3 (Taiaroa et al., 2020)).

By visual inspection, Caco-2 cells appear hardly affected by the infection; whereas, Calu-3 clearly show signs of impaired growth and cell death at 24 hr post infection (hpi), particularly when infected with SARS-CoV-2 (Figure S1I). Data from that time point, including e.g. the level of viral RNA (Figure 1A) should be therefore interpreted with caution. Taken together, we show that the three infected cell lines show distinct responses in respect to the course of SARS-CoV/-2 infection.

### SARS-CoV-2 infection leads to a two-fold stronger induction of ISGs compared to SARS-CoV

**Figure 2:**
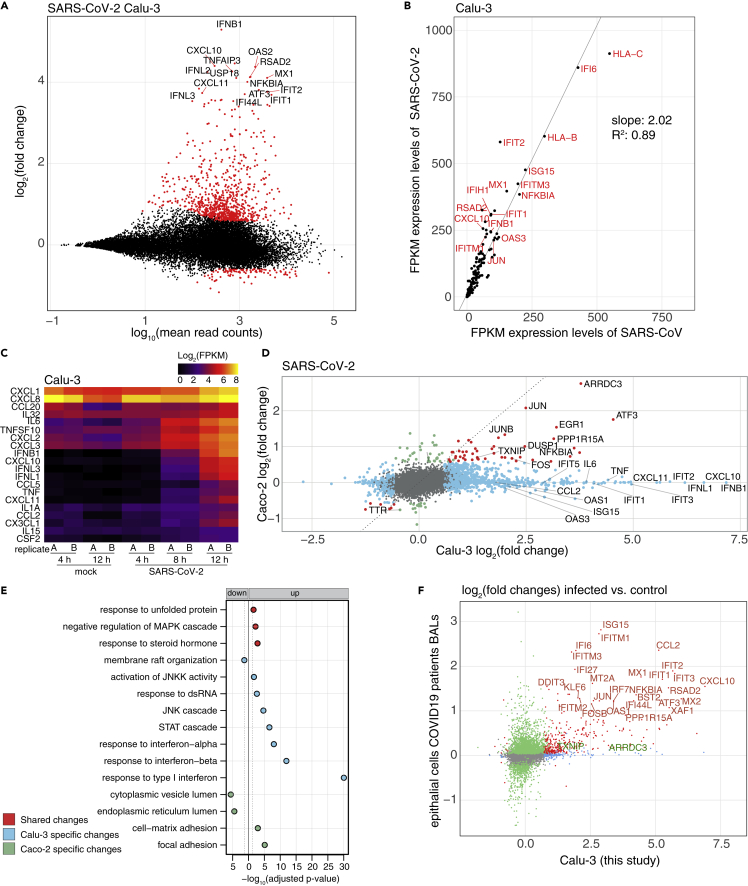
Dissection of the transcriptional response to SARS-CoV/-2 infection (A) Mean average vs. log_2_ fold change plot of differentially expressed (DE) genes at the mRNA level of Calu-3 cells infected with SARS-CoV-2 (12 hpi). X axis depicts log_10_ of mean counts associated with a gene. Y axis depicts mRNA log_2_ fold changes. Significant differentially expressed genes (red dots) are defined as those with an absolute log_2_ fold change of greater than 0.58 and a Benjamini-Hochberg corrected adjusted p value > 0.05. Selected outliers are labeled. (B) Expression values (FPKM) of genes significantly induced in cells infected with either virus at 12 hpi in Calu-3 cells. Values represent averages of the two replicates. (C) Expression values (FPKM) of differentially expressed cytokine genes in SARS-CoV-2 infected Calu-3 cells. Values of individual replicates are log_2_ transformed and represented as a heatmap. (D) log_2_-transformed fold changes of SARS-CoV-2 infected Calu-3 cells at 12 hpi vs. mock (horizontal axis) and Caco-2 cells 12 hpi vs. mock (vertical axis). Genes exhibiting significant changes in both cell lines are shown in red, significant changes only in Calu-3 cells in light blue, only in Caco-2 cells in light green. All other genes are shown in gray. Selected genes with significant fold changes are labeled. (E) Gene Ontology (GO) terms in a gene set enrichment analysis of cell lines infected by SARS-CoV-2. In blue are indicated enriched GO terms from differentially expressed genes in Calu-3 cells, in green from Caco-2 cells and in red from both cell lines. Adjusted p values were -log_10_-transformed. GO terms from downregulated genes are shown to the left, those from upregulated genes on the right of the solid line. The dotted line represents the cutoff value (p = 0.1). (F) Log_2_-transformed fold changes of SARS-CoV-2 infected Calu-3 cells of this study at 12 hpi (horizontal axis) and epithelial cells from severe COVID19 patients from Liao et al. (2020) (vertical axis). Genes exhibiting significant changes in both cells are shown in red, significant changes only in Calu-3 cells in light blue, only in patient cells in light green. All other genes are shown in gray. Selected genes with significant fold changes are labeled in red, and additional genes mentioned in the text (TXNIP and ARRDC3) in green.

To analyze changes in the host cell transcriptome, we compared virus- and cell line-dependent differences in gene expression. SARS-CoV-2 infection of Calu-3 cells, as expected from similar results for SARS-CoV infections (Yoshikawa et al., 2010), led to induction of a range of genes known to respond to viral infections, such as IFIT2, OAS2, or IFNB1 (Figure 2A) (Schoggins, 2019). This likely occurs through triggering of RNA sensors by incoming viral RNA via IRFs and NF-κB signaling (Schneider et al., 2014; Schoggins, 2019). Expression levels of these genes at 12 hpi were on average about twice as high in cells infected with SARS-CoV-2 compared to infection with SARS-CoV (Figure 2B), at similar amounts of viral RNAs present in the cells at 12 hpi (Figure 1A, right panel). This difference in the extent of the transcriptional response in SARS-CoV/-2 infections has also been observed in human airway epithelial cells (AECs) (V'kovski et al., 2020). Importantly, several cytokines (Carrasco Pro et al., 2018) are among the induced genes (Figure 2C), and which might be connected with pathologies such as the acute respiratory distress syndrome (ARDS) in CoV infections (Channappanavar and Perlman, 2017; Moore and June, 2020).

### Expression of arrestin-related domain-containing protein-3 and thioredoxin-interacting protein genes is induced independently of RNA sensing

Although the infection levels are comparable between Caco-2 and Calu-3 cells, based on the amount of intracellular viral RNA and virion yield (see above), the host transcriptome responses were markedly different (Figures 2D and S2A). Both cell lines showed an increase in expression of a number of genes typically activated in response to ER stress and MAP kinase activation (e.g. activating transcription factor 3, early growth response 1, EGR1; immediate-early response 3; protein phosphatase 1 regulatory subunit 15A (PPP1R15A) also known as growth arrest and DNA-damage-inducible 34; GADD34 (Lee et al., 2018)), with a corresponding enrichment of related GO terms in a gene set enrichment analysis (Figures 2E and S2B). Induction of genes regulated by IRFs was however absent in the Caco-2 cells employed here. One reason for this response might be the low expression of the pattern-recognition receptors IFIH1 and DDX58 (Figure S2C).

In addition, two genes, arrestin-related domain-containing protein-3 (ARRDC3) and thioredoxin-interacting protein (TXNIP), stood out among the few genes that were significantly upregulated upon infection with either viruses and in both cell lines (Figures 2D and S2A). Both genes encode proteins that are involved in regulation of signaling pathways (Aubry et al., 2009). ARRDC3 mediates G protein–coupled receptor lysosomal sorting and apoptosis-linked gene 2-interacting protein X (ALIX) ubiquitination (Dores et al., 2015). ALIX is a Lys63-specific polyubiquitin binding protein that functions in retrovirus budding and Dengue virus propagation (Dowlatshahi et al., 2012; Thepparit et al., 2019). TXNIP is involved in the regulation of glucose and lipid metabolism (Alhawiti et al., 2017) and has been shown to be involved in initiation and perpetuation of NLRP3 (nucleotide-binding domain and leucine-rich repeat and pyrin domain containing 3) inflammasome activation (Elliott and Sutterwala, 2015; Oslowski et al., 2012).

To conclude, most gene expression changes in response to SARS-CoV-2 infection are likely triggered by RNA sensors and/or ER stress. In H1299 cells, in which virus replication was very inefficient, we observed no consistent and notable differences in gene expression, as well as no alterations of the visual appearance (data not shown).

### Comparison of datat sets from *in vivo* and *in vitro* infections shows common and distinct responses to SARS-CoV-2 infections

To substantiate the relevance of the genes induced by the SARS-CoV-2 infection observed in the Calu-3 cells, we compared our data with various recently published data sets. This includes bronchoalveolar lavages (BALs) (Liao et al., 2020), as well as nasopharyngeal swabs (Chua et al., 2020) of patients infected with COVID-19 , and transcriptome studies of normal human bronchial epithelial (NHBE) cells, A549 cells with and without ACE2 expression, and also Calu-3 cells upon infection with SARS-CoV-2 (Blanco-Melo et al., 2020). (Table S2). For the BAL samples, differential gene expression was calculated for the epithelial cells (Figure S2D upper panel), from severe COVID-19 cases in comparison to the healthy control also used in the original paper (Figure 2F). Several genes were also found to be induced in the patient samples, such as several IFI/IFIT/IFITM genes, as well as the cytokines C-C motif chemokine ligand 5 (CCL2) and C-X-C motif chemokine ligand 9 (CXCL10), which were also upregulated in a BAL bulk RNA-seq analysis (Xiong et al., 2020). Remarkably, we did not observe induction of interferon genes, pro-inflammatory cytokines, and IL-6 in epithelial cells in patient samples. ARRDC3 and TXNIP, the two genes upregulated in both Calu-3 and Caco-2 cells, generally showed low expression with no induction in this analysis. However, when comparing expression levels within samples, we found an increase of ARRDC3 expression in a subset of patients compared to the healthy control, along with the consistent upregulation of the stress gene PPP1R15A (Figure S2D lower panel).

Within the epithelial cells in nasopharyngeal swabs, the highest expression values for the SARS-CoV-2 receptor ACE2 was found in secretory and ciliated cells (Chua et al., 2020), and we performed a similar analysis as above for these two cell types by comparing differential expression between the five control samples and the nineteen samples from infected patients (Figure S2E). The differentially expressed genes, which were commonly upregulated in patient samples, showed a smaller overlap with the Calu-3 cells compared to the BAL samples.

Infection of Calu-3 cells with SARS-CoV-2 showed strong reproducibility across experiments from our study (Figure S2F) and data from other laboratories (Figure S2G, upper panel). There were however considerable differences between Calu-3 and NHBE cells on one side (Figure S2G, lower panel), and A549 cells (Figure S2H) on the other side. As in the patient samples, interferon beta/lambda genes were not induced in NHBE cells (Figures 2F and S2G). On the other side, CXCL10 or IFIT2, which were induced in patient samples and Calu-3 cells, were not or not reproducibly induced in NHBE (Figure S2G) or A549 cells (Figure S2H). This however could also be due to variabilities in e.g. employed MOIs (Blanco-Melo et al., 2020). Taken together, the interferon and stress response observed in SARS-CoV-2 infected Calu-3 cells could be partially recapitulated in patient samples and other cell lines. Further investigations are warranted to better define appropriate model systems for studying the consequences of *in vivo* SARS-CoV-2 infections.

### MicroRNA miR-155-3p is expressed in SARS-CoV and SARS-CoV-2 infected cells

**Figure 3:**
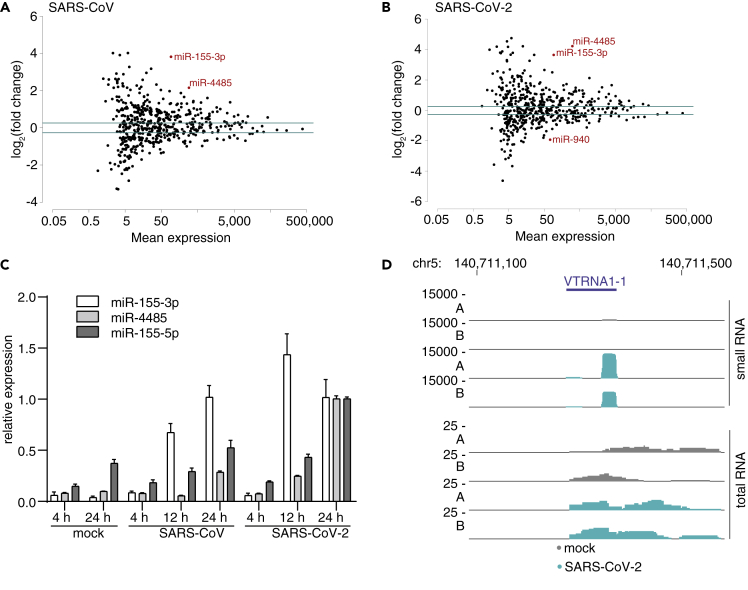
MicroRNA miR-155 and vaultRNA-derived miRNAs are induced by the infection (A and B) Scatterplots of miRNAs from of SARS-CoV (A) or SARS-CoV-2 (B) infected Calu-3 cells harvested at 12 hpi. Plotted are mean expression over log2(fold changes), normalized to mock infected (uninfected) cells. miRNAs found significantly differentially expressed are indicated in red. (C) Validation of the induction of the displayed microRNAs in Calu-3 cells at 12hpi using Taqman assays. Shown are average and standard deviations of three measurements from one biological replicates of the samples used for the sequencing in A and B, as relative expression normalized to 24 hpi SARS-CoV-2. (D) Coverage plots of the vaultRNA gene VTRNA1-1 of replicates A and B at 12hpi after infection of Calu-3 cells with or without SARS-CoV-2.

In addition to assessing mRNA changes, we have also profiled small RNAs expression changes in the context of Calu-3 infections. Both viruses triggered a close to 16-fold upregulation of miR-155-3p, the “star” form, and an almost 3-fold upregulation of miR-155-5p (Figures 3A, 3B, and S3A). Importantly, the primary miRNA precursor gene, miR-155 host gene (MIR155HG), was also upregulated in polyA-seq and total RNA-seq data by about 10-fold, suggesting that the increase of two miRNAs was primarily driven by transcription (Figure S3B). In addition, we found a significant upregulation of miR-4485. All observations were confirmed using a Taqman assay (Figure 3C). Of note, miR-4485 derives from the MTRNR2L8 locus, and it is unclear whether its expression pattern reflects its functionality or that of its host gene.

Interestingly, the miRNA profiling identified small RNAs mapping to vault RNA (VTRNA) genes to be induced by the infection (Figures 3D and S3C–S3E). The predominant lengths of these RNAs were 21 and 24 nt (Figure S3C). The function of vtRNAs has not been fully elucidated, but the mature VTRNA1-1 was recently discovered as a negative regulator of autophagy (Horos et al., 2019). VTRNA-derived sRNAs can be processed by DICER and bound by Argonaute proteins (Persson et al., 2009; Thomson et al., 2015) and could thus have a regulatory role in virus infected cells.

### scRNA-seq of infected Calu-3 cells shows expression of interferon genes only in a small subset of cells

**Figure 4:**
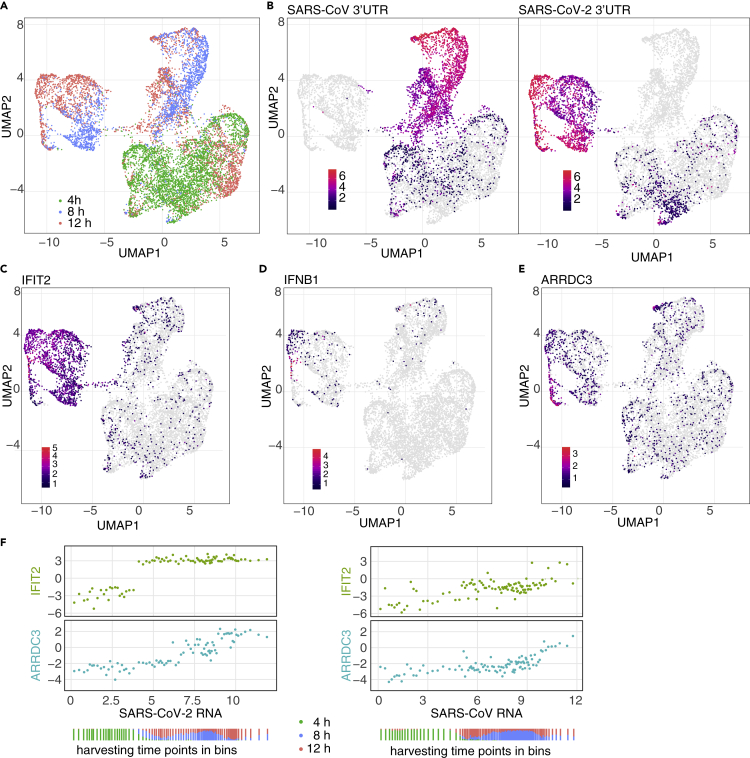
Single-cell RNA-sequencing shows different expression dynamics of genes induced by infection (A) Based on scRNA-seq data, Calu-3 cells were embedded into diffusion map space and, by Uniform Manifold Approximation and Projection (UMAP), projected using 20 diffusion components into two-dimensional space, and colored by harvesting time point. (B) Same projection as in A, with cells colored by SARS-CoV (left) and SARS-CoV-2 (right) 3′-UTR signal. (C–E) Same projection as in A, with cells colored by expression levels of IFIT2 (C), IFNB1 (D), and ARRDC3 mRNA (E). (F) Cells were sorted by the amount of viral RNA and binned. Horizontal axis for each panel represents relative, log_2_-transformed levels of SARS-CoV-2 RNA (left) or SARS-CoV (right) RNA per bin. The vertical axes in the panels represent relative, log_2_ transformed expression levels for IFIT2 (upper panel) or ARRDC3 (lower panel). Every dot represents a bin containing 50 cells. The distribution of the harvesting time points of the cells per bin is indicated below.

To assess gene expression changes on the level of individual infected Calu-3 cells, we performed scRNA-seq at different time points post infection for both SARS viruses. The data showed that cellular transcriptomes grouped by time point of infection (Figure 4A) and type of virus (Figure 4B). At 4 hpi, the number of cells bearing viral RNA was between 40% and 60% (Figure S4A, Table S4). At 8 hpi and 12 hpi, all cells contained viral RNA. The distribution of viral load (percentage of viral RNA per cell) was comparable for the two viruses and showed the expected increase from 4 hpi to the later time points after infection; however biological replicates showed some variability (Figures S4B and S4C). The amount of detected RNA molecules (UMI, unique molecular identifiers) was generally lower in cells with high levels of viral RNA (Figure S4D), indicative of some degree of host cell shutoff.

In agreement with the bulk RNA-seq data, we observed a strong increase of e.g. IFIT2 or OAS2 in infected cells (Figures 4C and S4E). Again, the induction of these genes was much stronger in the cells infected with SARS-CoV-2 compared to infection with SARS-CoV (Figures 4C and S4E).

In the bulk RNA-sequencing data, interferon beta (IFNB1; Figure 2A) was one of the most induced genes upon SARS-CoV-2 infection. In the scRNA-seq data, we found the expression of IFNB1 expression restricted to a small subset of infected cells (Figure 4D). As in bulk RNA-sequencing, we observed expression of ARRDC3 in cells infected with either viruses. ARRDC3 (Figure 4E), as well as PPP1R15A (Figure S4F), were highly expressed in cells with the highest levels of viral RNA. For SARS-CoV infection, we observed similar effects, however at overall lower levels and in very small numbers of cells (Figure 4D, top part of the plots).

To confirm the induction of the microRNA mir-155 described in the previous section, we investigated the expression of its host gene, MIR155HG. Although it is expressed at only low levels, it resembled the expression pattern of IFIT2 and OAS2 genes (compare Figure S4G with Figure S4E), i.e. induction in cells containing SARS-CoV-2 RNA and high levels of SARS-CoV RNA.

To relate host gene expression to the accumulation of viral RNAs, cells were ordered by an increasing amount of viral RNA and arranged into bins of 50 cells. This was done to reduce noise due to detection dropout events. The correlation with viral RNA over all bins was then calculated for both viruses, indicating a particularly strong relationship for the amount of viral RNA the expression of, among others, ARRDC3 (Figure S4K). For IFIT2 and ARRDC3, the expression level per bin was then plotted against the amount of viral RNA (Figure 4F, left). For IFIT2, a stepwise expression increase between 4 hpi and 8 hpi was observed, with the expression levels afterward being independent of the amount of SARS-CoV-2 RNA. ARRDC3 mRNA transcript levels however increased gradually with the accumulation of viral RNA. For SARS-CoV, this transcriptional induction was observed only at much higher levels of viral RNA were (Figure 4F, right).

In order to identify genes co-regulated with IFNB1, we performed a correlation analysis of cells binned by increasing IFNB1 and ARRDC3 expression (Figure S4L). Using this approach, we found a putative co-regulation of the four interferon lambda genes (IFNL1-4), the chemokine genes, CXCL9 and CCL5, and the cholesterol-25-hydroxylase gene. This enzyme, as well as its product 25-hydroxycholesterol, has been shown to act against a range of viruses (Liu et al., 2013). In addition, two other genes were found in this group, the sodium voltage-gated channel alpha subunit 3 gene (SCN3A) (Figure S4M) and the dual oxidase 1 gene (DUOX1) (Figure S4N). Although SCN3A has previously not been described in the context of virus infections, DUOX1 appears to promote the innate immune defense to pathogens via the production of reactive oxygen species (De Deken et al., 2014).

### RNA velocity reveals transient induction of interferon genes and temporal resolution of NF-κB signaling

**Figure 5:**
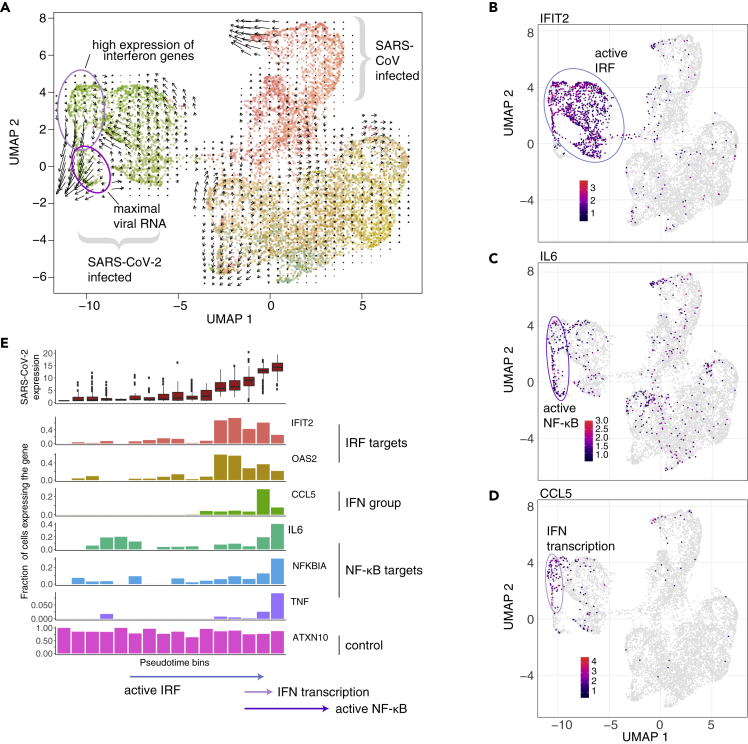
Interferon genes and NF-κB target genes are expressed in small subsets of cells (A) Embedding as in Figure 4. Areas of interest are marked by colored ovals and labeled. Arrows represent trajectories based on RNA velocity, signal strength is represented by the length of the arrows. (B–D) Projection as in A, but colored by intron counts of IFIT2 (B), IL6 (C), and CCL5 (D). Areas of interest are marked by colored ovals and labeled. (E) Cells were grouped into pseudotime bins. Plotted are, from top to bottom, the amount of SARS-CoV-2 viral RNA, and the percentage of cells with intron counts for the indicated genes.

To better understand the nature of interferon beta/lambda gene induction in the context of SARS-CoV-2 infection, we applied RNA velocity, which uses sequencing reads originating from introns to measure the amount of nascent mRNA (La Manno et al., 2018). This analysis inferred a temporal trajectory, represented by the black arrows in Figure 5A, with longer arrows indicating a stronger signal. For the SARS-CoV-2 infected cells, the directionality was particularly strong from cells expressing interferon and interferon-correlated genes to the cells with maximal amount of viral RNA but not expressing interferon genes (Figure 5A). This finding suggests that induction of interferon genes is short and transient during the viral replication. A similar effect was seen for SARS-CoV infection. We observed that target genes of IRFs such as IFIT2, IFIT1, or OAS2 (Grandvaux et al., 2002) show high intron counts in most of the cells infected with SARS-CoV-2, except for a subpopulation (Figure 5B underneath the blue oval, S5B). This subpopulation, however, showed intron counts for NF-κB target genes such as interleukin 6 (IL6), tumor necrosis factor (TNF) or NF-κB inhibitor alpha (NFKBIA) (Figure 5C bottom part of the violet oval, S5B). Since the IFNB1 gene is devoid of introns and intron counts from IFNL1-4 were not detected, we used CCL5 as a reference for this group of genes (compare Figure S5A with Figure 4D, middle panel). Interestingly, we found that its transcriptional activity is restricted to a small subset of cells (Figure 5D) at the intersection of IRF and NF-κB activity.

To identify the temporal evolution of changes in gene expression, we have looked at the patterns of host and viral gene expression in the principled space of a diffusion map (Angerer et al., 2016; Haghverdi et al., 2016). As expected, the cells infected with the two viruses separated on two branches according to the main diffusion components (Figure S5C). Especially for SARS-CoV-2, there was a noticeable population of cells (Figure S5C, circled by a violet oval) marked by high levels of both viral RNA and IL6 mRNA, whereas the highest levels of IFNB1 and IFIT2 mRNA were in distinct subpopulations, indicating the importance of higher order diffusion components for the proper temporal stratification (Figure S5C, pink and blue ovals). To corroborate the velocity analysis shown in Figure 5A, we projected the velocity data on an alternative embedding, initialized with diffusion pseuodotime, i.e. reflecting cell-to-cell transition probabilities (Figure S5D). In there, we observed the same biological effect as in Figure 5, namely that transient induction of interferon genes is followed by expression of NF-κB target genes.

For a comprehensive analysis, we have binned SARS-CoV-2 infected cells by applying the Louvain algorithm on the top fifty diffusion components and sorted the resulting bins by median SARS-CoV-2 load (Figure 5E, top row). Per bin, the intron counts of the infection-induced genes IFIT2, OAS2, CCL5, IL6, NFKBIA, and TNF are shown, along with ATXN10 as control (Figure 5E). The temporal ordering suggests that IRF-regulated genes (OAS2, IFIT2) were transcribed before NF-κB target genes (IL6, NFKBIA, TNF), and likely only during a relatively short time window, both pathways were active and drove transcription of interferon and related genes (CCL5).

In addition, we performed the RNA velocity analysis on an embedding of the Calu-3 cells calculated without viral genes, which is now driven by the genes induced by the infection such as IFIT2 or ARRDC3, leading to a convergence of highly infected cells independent of the virus (Figure S5E).

Interestingly, we also observed a minor increase in intronic counts for ACE2 transcripts in SARS-CoV-2 infected cells (Figures S5F and S5G), suggesting a transcriptional activation of the viral receptor gene during infection, as observed recently (Ziegler et al., 2020).

### scRNA-seq of SARS-CoV and SARS-CoV-2 infected H1299 reveals a potential involvement of HSP90AA1 in the progression of infection

As shown above, transcriptional changes in bulk and scRNA-seq data from Calu-3 infected cells were dominated by the interferon response. In order to detect more subtle alterations in the cellular transcriptomes, we applied the scRNA-seq likewise to SARS-CoV/-2 infected H1299 cells, which are only partially permissive to the infection.

**Figure 6:**
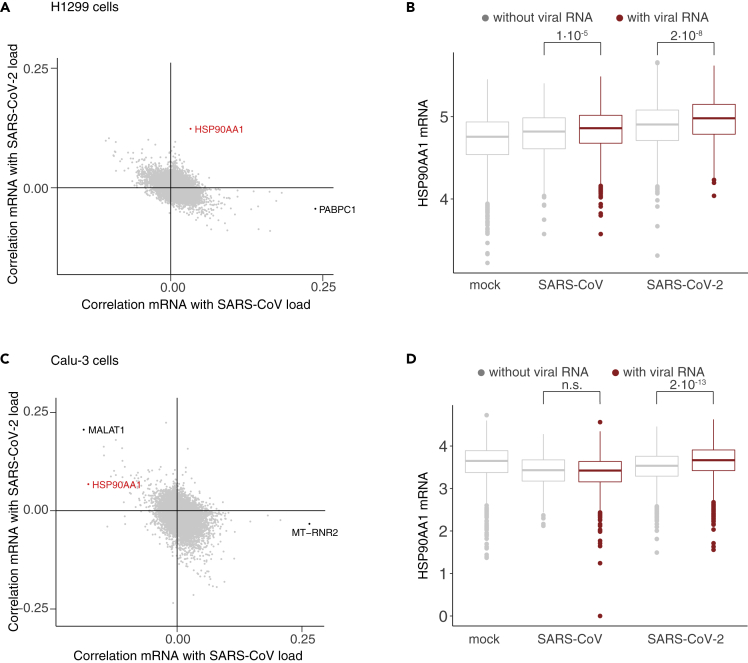
HSP90AA1 is deregulated in SARS-CoV-2 infected cells (A) Correlation of gene expression values with the amount of SARS-CoV RNA (horizontal axis) and SARS-CoV-2 RNA (vertical axis) in the H1299 scRNA-seq data. (B) Distribution of HSP90AA1 mRNA expression in H1299 single cell samples. For SARS-CoV and SARS-CoV-2 samples, cells were group by presence of viral RNAs. To calculate p values, the Kruskal-Wallis test was used (p.val < 1 × 10^−5^, with 3 degrees of freedom), followed by post-hoc comparison of classes using the Dunn test with Bonferroni correction. Non-significant (n.s.) indicates p values larger than 1 × 10^−5^. (C and D) As in A, B, but for Calu-3 (4 hpi) samples.

Despite the overall low amount of viral RNA in infected H1299 cells (Figure 1A), the percentage of cells bearing viral transcripts was unexpectedly high (Figure S6A, Table S4), indicating that virions indeed are able to enter the cells. As seen in the bulk RNA-seq, transcriptional changes were subtle, and cells do not group into discrete clusters (Figures S6B–S6D). When correlating individual genes with the amount of viral RNA over cells, we found a positive correlation with HSP90 alpha family class A member 1 (HSP90AA1) with the amount of SARS-CoV-2 RNA but not SARS-CoV (Figure 6A). When comparing expression within samples, we observed higher levels of HSP90AA1 mRNA in cells with SARS-CoV-2 viral RNA compared to those without (Figure 6B). To exclude that this would not be a general effect for highly expressed transcripts, we performed the same analysis for GAPDH, which remained unchanged in virus-positive cells (Figure S6E).

For the Calu-3 cells, we found a similar HSP90AA1 expression pattern in the data from the 4 hpi time point (Figures 6C and 6D). However, in this analysis, we observed, like for HSP90AA1, a positive correlation of MALAT1 RNA levels with the amount of viral RNAs in the cells (Figures 6C and S6F). Previously, MALAT1 was shown to be upregulated by the UPR during flavivirus infection (Bhattacharyya and Vrati, 2015).

In order to investigate HSP90AA1 mRNA deregulation in patients infected with COVID-19, we re-examined the BAL scRNA-seq (Figure 2F). We observed that in a cluster of epithelial cells, with a substantial number of cells containing viral RNA, HSP90AA1 was among few genes that were deregulated in cells with viral transcripts compared to those without (Figure S6G).

Overall, we found HSP90AA1 deregulation in various datasets, suggesting a role for HSP90AA1 in SARS-CoV-2 infection.

### Inhibition of HSP90 reduces viral yield and expression of cytokine genes

**Figure 7:**
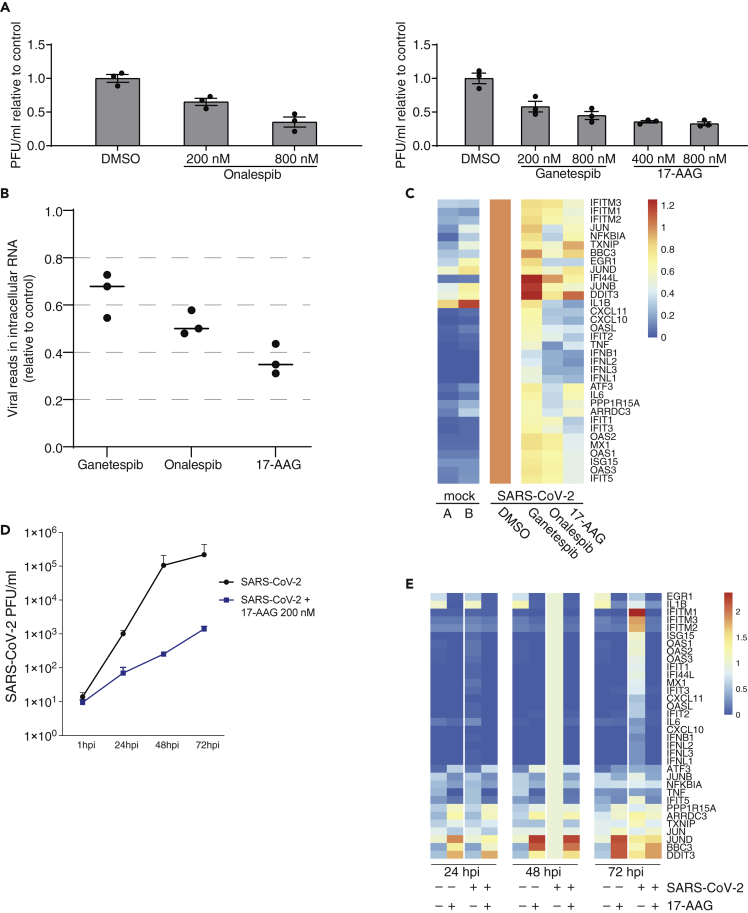
HSP90 inhibitors treatment reduce SARS-CoV-2 replication and induction of pro-inflammatory cytokines (A) Fold change of infectious plaque-forming units (PFUs) in supernatants of Calu-3 cells infected with SARS-CoV-2 with the indicated treatment 16 hpi. After viral adsorption for one hour, cells were washed and supplied with conditioned medium containing DMSO, as solvent control, or indicated concentrations of Onalespib, Ganetespib, or 17-AAG. Experiment performed in triplicates, and error bars represent standard deviations. (B) Percentage of viral sequencing reads of all reads in Calu-3 cells after 800 nM of indicated inhibitor treatment 16 hpi. (C) Heatmap of selected induced genes normalized to infection in Calu-3 cells without treatment. (D) Infectious viral particles in the supernatant at the indicated timepoints post infections of primary human airway epithelial cells (AECs) treated with either DMSO (control, black) or 200 nM 17-AAG (blue). (E) Heatmap of selected induced genes in AECs normalized to the infection sample treated with DMSO of the same timepoints (average of normalized expression values/FPKM).

The involvement of HSP90AA1, a highly-conserved molecular chaperone, in viral infections has since a long time been discussed to be involved in the infection of a range of viruses (Geller et al., 2012). In order to explore the effect of HSP90 on SARS-CoV-2 replication in Calu-3 cells, we applied the HSP90 inhibitors Onalespib, Ganetespib, and 17-(Allylamino)-17-demethoxygeldanamycin (17-AAG) to cells one hour after viral absorption and measured virus yield and RNA in the supernatant at 16 hpi. At inhibitor concentrations of 800 nM, viral yield was reduced by about 50%–70% (Figure 7A). Cell viability was not impaired using the indicated inhibitor concentrations (Figure S7A). In order to assess the production of intracellular viral RNA and changes in host gene expression, we performed bulk RNA-sequencing of uninfected cells, as well as DMSO (solvent control) and HSP90 inhibitor treated infected cells. Intracellular viral RNA was reduced comparable to viral yield (Figure 7B). The treatment of cells with HSP90 inhibitors dampened the upregulation particularly of pro-inflammatory cytokines, including IL-6, CXCL10, and CXCL11 (Figures 7C and S7B). In summary, inhibition of HSP90 reduced both the viral replication and the induction of the inflammation response.

To assess the effect of HSP90 inhibition in primary human cells, we used AEC differentiated *in vitro* from human lung tissue obtained from pneumonectomy specimens (Imai-Matsushima et al., 2018). Again, SARS-CoV-2 replication was considerably slower upon treatment with the HSP90 inhibitor 17-AAG at 200 nM (Figure 7D). Under these conditions, cell viability was not impaired (Figure S7C). We again assessed changes in host gene expression by RNA-sequencing, and observed a reduction of mRNA levels of various genes induced by the infection, including the cytokines CXCL10 and CXCL11 (Figure 7E). As observed in Calu-3 cells, chemical inhibition of HSP90 in infected human primary cells reduced viral replication and the induction of the inflammatory response, suggesting that 17-AAG or other HSP90 inhibitors already applied in clinical trials could be used for the therapeutic interventions in the treatment of COVID-19.

## Discussion

We performed gene expression profiling of three different human cell lines infected with SARS-CoV and SARS-CoV-2 at bulk and single-cell level. We show a particularly strong induction of a range of genes commonly induced by virus infections in Calu-3 cells, including cytokines, by SARS-CoV-2 in both bulk and scRNA-seq experiments. For various CoVs, a range of mechanisms that interfere with interferon signaling have been reported (Kindler et al., 2016). For SARS-CoV, it was shown that ORF6 inhibits signal transducer and activator of transcription (STAT) signaling (Frieman et al., 2007) and that IRF3 activity is impaired (Spiegel et al., 2005). Since RNA levels per cell (Figure S4A) and on the population level were comparable (Figure 1A), it is tempting to speculate that such mechanisms could be less efficient in SARS-CoV-2 compared to SARS-CoV.

By comparing the transcriptional response of Calu-3 cells to that of Caco-2 cells, which might have reduced capability to sense incoming RNA (Figure S1A), we identify genes induced independently of the RNA sensing system, such as the ER stress marker PPP1R15A. We also observed upregulation of the genes TXNIP and ARRDC3. Both are involved in signaling processes, and further investigations into their role in SARS-CoV-2 infection are warranted.

On the other hand, in H1299 cells, viral replication was very inefficient both in terms of extracellular viral yield and intracellular viral RNA. The percentage of cells with viral reads in the single-cell experiment (Figure S6A) is relatively high, but in contrast to the Calu-3 cells not consistently increasing over time. Whether this means that virus particles do not productively enter into the cytoplasm, or suppression of replication in these cells, cannot be answered from our data.

Small RNA profiling indicated an increased expression of miR-155 in the infected cells. This miRNA has been associated with various virus infections (Badry et al., 2020; Dickey et al., 2017; Gottwein, 2013; Zeng et al., 2015). miR-155-3p is also a well-known regulator of immune cells, in particular T cell differentiation (Mehta and Baltimore, 2016; Thai et al., 2007). Involvement of this miRNA in the regulation of innate immunity has also been reported (Zhou et al., 2010). Recently, miR-155-5p expression was shown to be induced in mice infected with influenza A virus (Woods et al., 2020). Importantly, in this study, lung injury by ARDS was attenuated by deletion of miR-155, making this miRNA a potential therapeutic target in the context of COVID-19.

The scRNA-seq experiments provided a rich dataset to analyze host cell expression changes in response to the infection. Surprisingly, the percentage of cells containing viral RNA was much higher than expected based on the MOI used for the infection experiments. A possible explanation for this observation could be viral spreading by cell-to-cell fusions, facilitated by the S protein on the cell surface (Buchrieser et al., 2020; Masters, 2006). Furthermore, the analysis of the scRNA-seq data of infected Calu-3 cells indicated a sequential activation of IRF and NF-κB target genes, and in particular, a putatively strong, but transient, induction of interferon genes. This could be due to a relatively short time window during the progression of infection, in which both IRF and NF-kB are sufficiently activated to trigger interferon gene transcription (Czerkies et al., 2018; Iwanaszko and Kimmel, 2015).

Concomitantly with the interferon induction, we observed a small increase in transcription of ACE2 mRNA in cells infected with SARS-CoV-2. Changes in ACE2 transcript levels in the context of interferon treatment and CoV infection have been described before (Gralinski and Baric, 2015; Ziegler et al., 2020). However, further studies are needed to explore whether mRNA changes are reflected on the protein level, particularly since a recent report indicates that the transcriptionally upregulated ACE2 mRNA does not yield functional protein (Onabajo et al., 2020). Although the transcriptional induction (RNA velocity) was detectable, changes in mature mRNA levels were moderate in comparison to IRF-driven genes such as the IFIT family.

Cell line and cell culture-based infections allow detailed analyses of molecular changes during infections, since perturbations such as genetic manipulations or compound treatments can be straightforwardly applied. However, they come with limitations and focus on isolated cells apart from tissues or organisms. This involves e.g. the production of cytokines, which was described to be connected to COVID-19 pathogenesis (Blanco-Melo et al., 2020; Channappanavar and Perlman, 2017; Moore and June, 2020). Here, infected Calu-3 cells showed a strong increase in expression of a number of chemokines, interferons, and pro-inflammatory cytokines, whereas A549 and NHBE cells responded with an induction of pro-inflammatory cytokines (Figure S2). However, in epithelial cells from BALs of patients infected with COVID-19, IL-6 or TNF mRNAs were barely detectable. Since these and interferon genes were transcriptionally activated only in small subsets of Calu-3 cells, this could represent an unlikely physiological cellular state. Overall, how to best match processes happening in the human body using *in vitro* models will require further investigations using a range of models and detailed matching with patient data (Butler et al., 2018; Duan et al., 2020; Lamers et al., 2020; Stanifer et al., 2020).

CoVs induce ER stress and activate the UPR in infected cells (Fung et al., 2016; Versteeg et al., 2007). We observed transient deregulation of the stress-responsive HSP gene HSP90AA1 (Zuehlke et al., 2015), in both the “slow-motion” infection model, H1299 cells, and in Calu-3 cells (4 hpi), and in subsets of epithelial cells in patient samples. HSP90 modulates UPR by stabilizing the ER stress sensor transmembrane kinases IRE1α (Marcu et al., 2002). Inhibition of the HSP90 has previously been shown to slow down the replication of several viruses (Gao et al., 2014; Geller et al., 2012; Katoh et al., 2017; Li et al., 2004). The reduction of SARS-CoV-2 growth by HSP90 inhibition was also proposed based on a computational analysis of patient RNA sequencing data (Sultan et al., 2020). Here, we show that inhibition of HSP90 by three different compounds at high nanomolar concentrations can reduce virus replication in an *in vitro* infection model. Interestingly, IFIT2 mRNA levels seemed unaffected by HSP90 inhibition, supporting the “on-off-switch” independent of the amount of viral RNA observed in the scRNA-seq data. In addition, mRNA expression of the pro-inflammatory cytokines TNF and IL1B, which are implied in the progression of COVID-19 (Hirano and Murakami, 2020), were strongly reduced. This repression is likely due to the requirement of HSP90 for constitutive and inducible IKK and NF-κB activation (Broemer et al., 2004). Interestingly, in addition to the already described activities, HSP90 inhibitors were shown to exert barrier protective effects on pulmonary arterial endothelial cells and were suggested to have useful therapeutic value in ARDS and other pulmonary inflammatory diseases (Antonov et al., 2008). Several HSP90 inhibitors have been in clinical development as anticancer agents (Gao et al., 2019) and have advanced to phase 2 and 3 clinical trials. Some of these compounds could be readily available to become part of a therapeutic strategy for COVID-19 by possibly inhibiting SARS-COV-2 replication, reducing inflammation and protecting endothelial barrier function.

### Limitations of the study

The major limitation of the study is the usage of epithelial cell lines, which might respond differently compared to epithelial cells in an organism, and intense data comparisons are necessary to define physiologically relevant effects. Particularly, the high levels of viral RNA in some cells, which lead to induction of interferon and NF-kB target genes, might not be reached in epithelial cells in infected organisms (Nouailles et al., 2020). For the Hsp90 inhibitor, the effects described here need to be confirmed in animals and humans before clinical relevance can be attributed to this compound class.

### Resource availability

#### Lead contact

Markus Landthaler, markus.landthaler@mdc-berlin.de.

#### Materials availability

No new reagents were created in this study.

#### Data and code availability

Raw sequencing data as well as count tables are for bulk and scRNA-seq experiments from cell lines are available at the Gene Expression Omnibus database (GEO), identifier GSE148729 (GEO: https://www.ncbi.nlm.nih.gov/geo/query/acc.cgi?acc=GSE148729). Supporting files are available on zenodo (Zen: Zenodo: https://zenodo.org/record/4031204#.YCpK_S1h1Bw).

## Methods

All methods can be found in the accompanying Transparent methods supplemental file.

## Supplemental information

Document S1. Transparent methods, Figures S1–S7, and Tables S1, S2, and S4

Table S3. Junction spanning-reads from viral RNA, related to Figure 1

Data S1. Readcounts per gene for the airway epithelial cell sequencing data, related to Figure 7
