# Supplementary material for: Transcriptomic profiling of SARS-CoV-2 infected human cell lines identifies HSP90 as target for COVID-19 therapy
Source: iScience. 2021 Feb 6;24(3):102151. doi: 10.1016/j.isci.2021.102151 (PMC7866843; doi:10.1016/j.isci.2021.102151)
Supplement: Document S1. Transparent methods, Figures S1–S7, and Tables S1, S2, and S4 [file mmc1.pdf]

iScience, Volume *24*

Supplemental Information

Transcriptomic profiling of SARS-CoV-2 infected human cell lines identifies HSP90 as target for COVID-19 therapy

Emanuel Wyler, Kirstin Mösbauer, Vedran Franke, Asija Diag, Lina Theresa Gottula, Roberto Arsiè, Filippos Klironomos, David Koppstein, Katja Hönzke, Salah Ayoub, Christopher Buccitelli, Karen Hoffmann, Anja Richter, Ivano Legnini, Andranik Ivanov, Tommaso Mari, Simone Del Giudice, Jan Papies, Samantha Praktiknjo, Thomas F. Meyer, Marcel Alexander Müller, Daniela Niemeyer, Andreas Hocke, Matthias Selbach, Altuna Akalin, Nikolaus Rajewsky, Christian Drosten, and Markus Landthaler

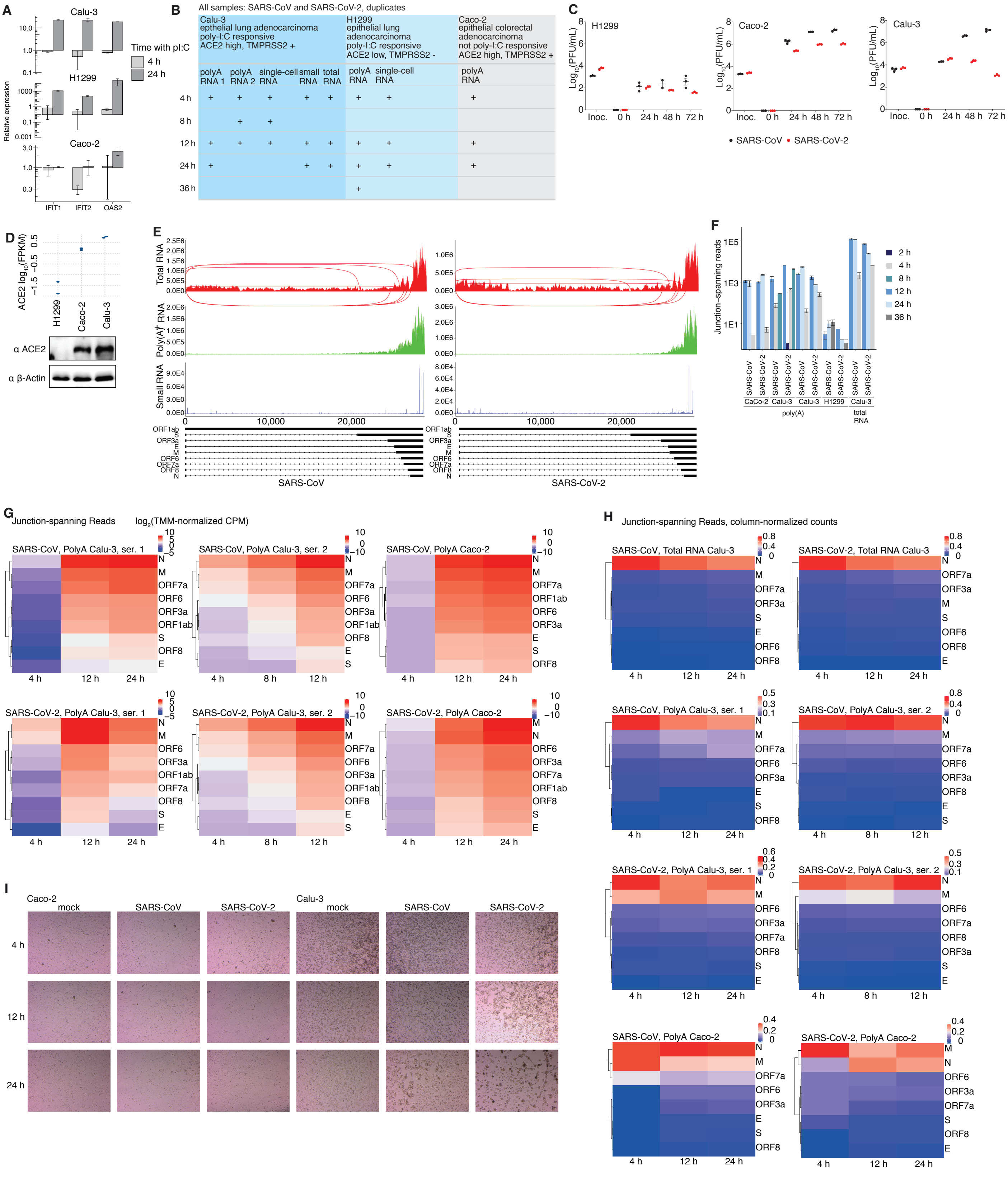

Figure S1. Different permissiveness of SARS-CoV/-2 infection in cell lines, Related to Figure 1

A, Relative quantification (RQ) of responsiveness to dsRNA of the cell lines as tested by RT-qPCR of three ISGs upon transfection of poly-I:C.

B, Overview of experimental set up and collected datasets. Calu-3 cells turned out to be the most suitable cell line.

C, Growth kinetics of SARS-CoV and SARS-CoV-2 in the different cell lines (MOI 0.01). Log10 of plaque forming units (PFU) of the inoculum (inoc.) and different hours post infection are plotted.

D, Expression values of the ACE2 mRNA in the polyA RNA-seq mock samples (upper part) and protein expression assessed by Western blot analysis with specific antibodies of indicated cell lines (lower part). H1299 cells showed neither mRNA nor protein expression of ACE2.

E, Coverage across the viral genome merged across all datasets for total RNA-seq, poly(A)+ RNA-seq, and small RNA-seq data. The top eight junctions supported by split reads are plotted in “sashimi” style for the total RNA-seq.

F, Barplot of junction-spanning reads from poly(A) and total RNAseq at indicated time point of different cell lines and series.

G and H, Heatmaps of canonical junction-spanning reads, averaged across biological replicates per time point, expressed in TMM-normalized counts per million (G), or relative counts per time point (H). ORF1ab levels are estimated by counting contiguous reads mapping to the leader junction site.

I, Phase-contrast microscopy images of with either virus infected Caco-2 and Calu-3 cells at indicated time points. CaCo-2 cells appear hardly affected by the infection; whereas, Calu-3 clearly show signs of cell death at 24 hours post infection (hpi), particularly when infected with SARS-CoV-2.

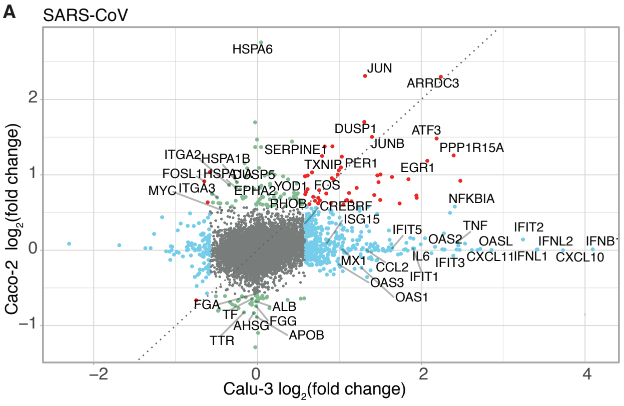

Figure S2. Dissection of the transcriptional response to SARS-CoV/-2 infection, Related to Figure 2

A, log2-transformed fold changes of SARS-CoV infected Calu-3 cells at 12 hpi vs. mock (horizontal axis) and Caco-2 cells 12 hpi vs. mock (vertical axis). Genes exhibiting significant changes in both cell lines are shown in red, significant changes only in Calu-3 cells in light blue, only in Caco-2 cells in light green. All other genes are shown in grey. Selected genes with significant fold changes are labeled.

B, Gene Ontology (GO) terms in a gene set enrichment analysis. Enriched GO terms from genes differentially in both Calu-3 and Caco-2 cells infected with SARS-CoV are shown in red, from differentially expressed in Calu-3 only in blue, and from differentially expressed in Caco-2 only in green. Adjusted p-values were -log10-transformed. GO terms from downregulated genes are shown to the left, those from upregulated genes on the right of the solid line. The dotted line represents the cutoff value.

C, Expression values (FPKM from polyA(+) RNA-seq) of the RNA sensors IFIH1 and DDX58 in mock-infected Calu-3 and Caco-2 cells.

D, upper panel: Data from BAL scRNA-seq patient samples from Liao et al. 2020 obtained via the GEO database, accession number GSE145926, analyzed together with the healthy control HC4 from GEO accesstion number GSM3660650. Cells are colored by cluster in a two-dimensional UMAP projection. Epithelial cells were identified based on TPPP3 and KRT18 marker gene expression (not shown). Lower panel: expression values of the indicated genes per cluster are shown as box plots.

E, Log2-transformed fold changes of SARS-CoV-2 infected Calu-3 cells of this study at 12 hpi (horizontal axis) and ciliated (left) and secretory (right) cells from COVID19 patients from Chua et al., 2020 (vertical axis). Genes exhibiting significant changes in both cells are shown in red, significant changes only in Calu-3 cells in light blue, only in patient cells in light green. All other genes are shown in grey. Selected genes with significant fold changes are labeled in red, and additional genes mentioned in the text (TXNIP, PPP1R15A, ARRDC3) in green.

F, Comparison of log2 transformed fold changes in Calu-3 in the two experiments from this study 12 hpi (series 1 and series 2).

G-H, Comparison of log2 transformed fold changes in Calu-3 from series 2 of this study with various series of SARS-CoV-2 infected cells from Blanco-Melo et al. F, comparison with Calu-3 cells (series 7). G, Comparison with NHBE cells 12 hpi (series 1). H, comparison with A549 cells (series 2 and series 3 without, series 6 with ACE2 transduction).

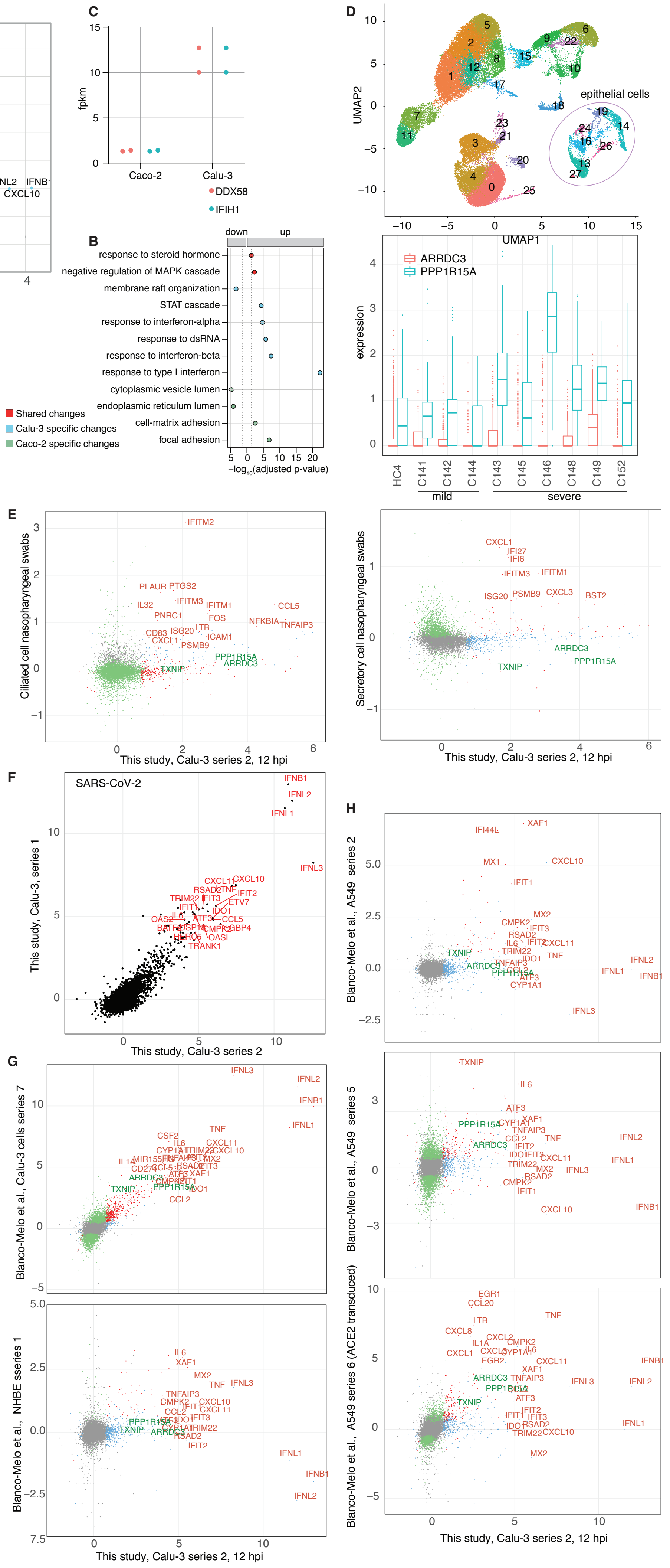

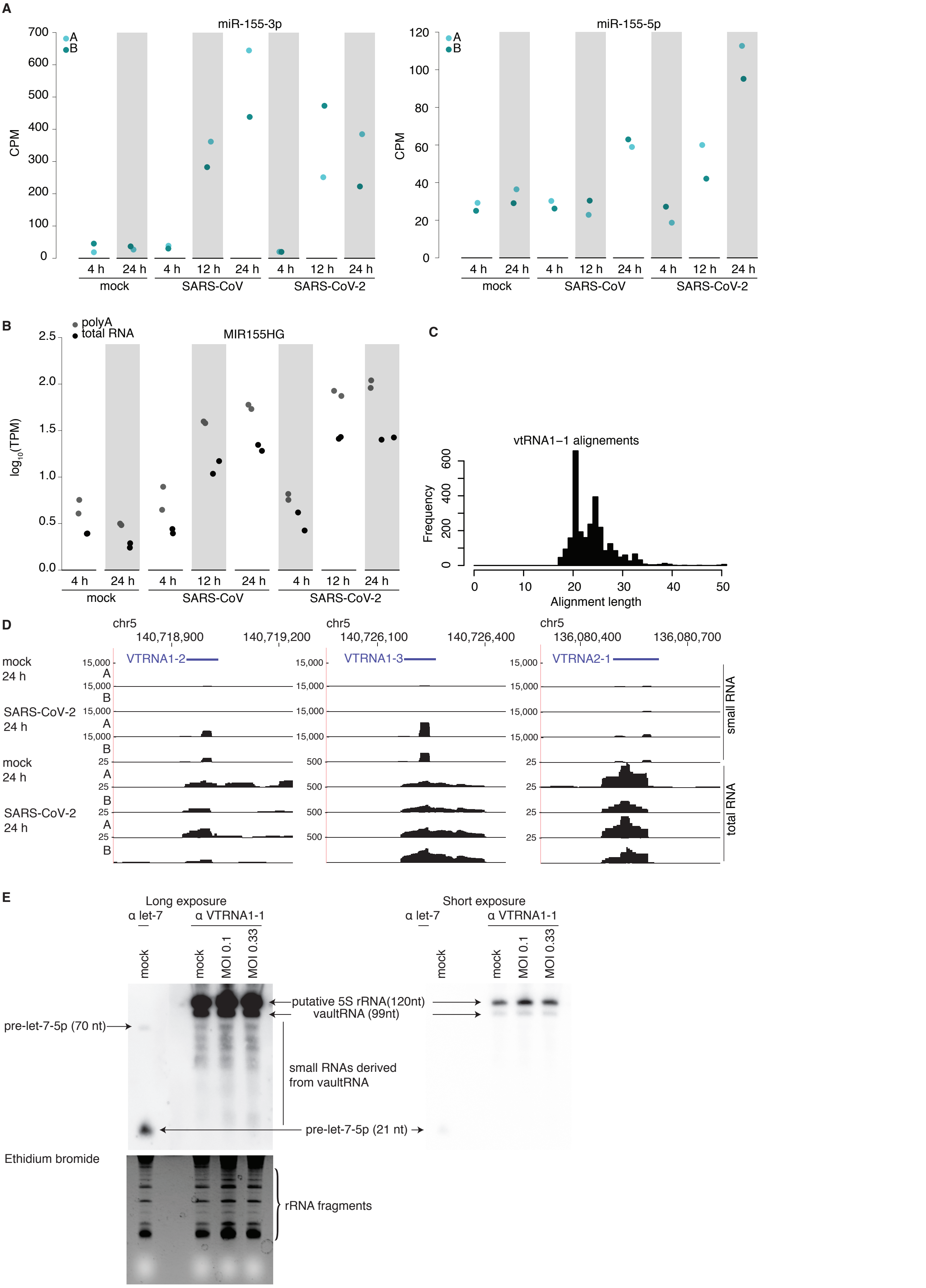

Figure S3. MicroRNA miR-155 and vaultRNA-derived miRNAs are induced by the infection, Related to Figure 3

A, Normalized counts (counts per million) of miR-155-3p (left panel) and miR-155-5p (right panel), colored by replicate.

B, Log10 miRNA-155 host gene transcripts per million in samples measured by polyA- or total RNAseq.

C, length distribution of small RNAs aligning to the VTRNA1-1 locus

D, coverage plots of the three vaultRNA genes VTRNA1-2, VTRNA1-3, and VTRNA2-1.

E, validation of small RNAs from the VTRNA1-1 locus using Northern blotting of SARS-CoV-2 infected Calu-3 cells 24 hpi. Input 5 μg total RNA. Left panel: probing with probes recognizing let-7-5p (left lane) and vtRNAs (three lanes on the right. Predicted sizes are indicated. The strong band above the vtRNA likely represents 5S/5.8S rRNA. Right panel: same as left panel, but short exposure to visualize vtRNA1-1 levels. Botttom: Ethidium bromide staining as loading control with tRNA with predicted size indicated.

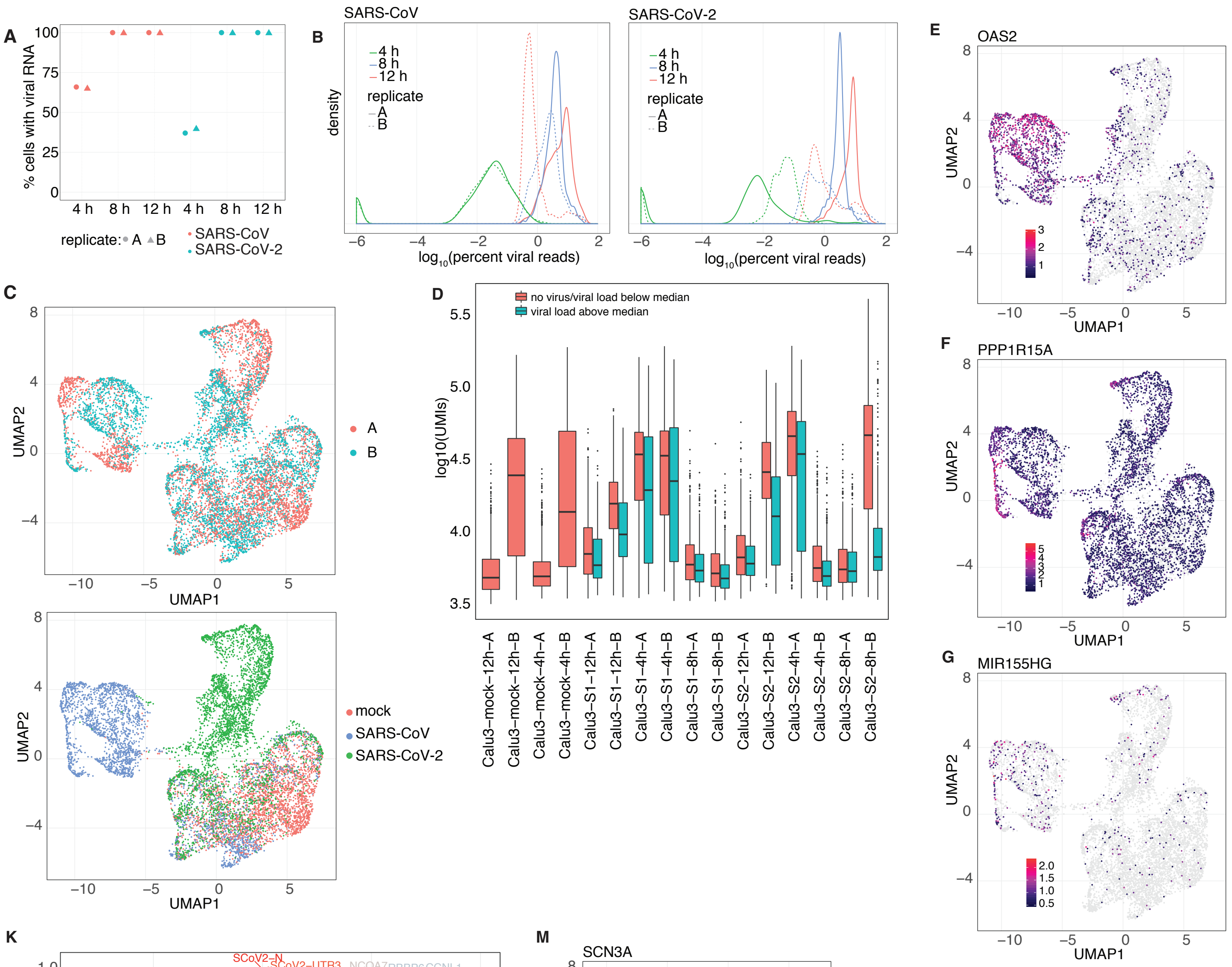

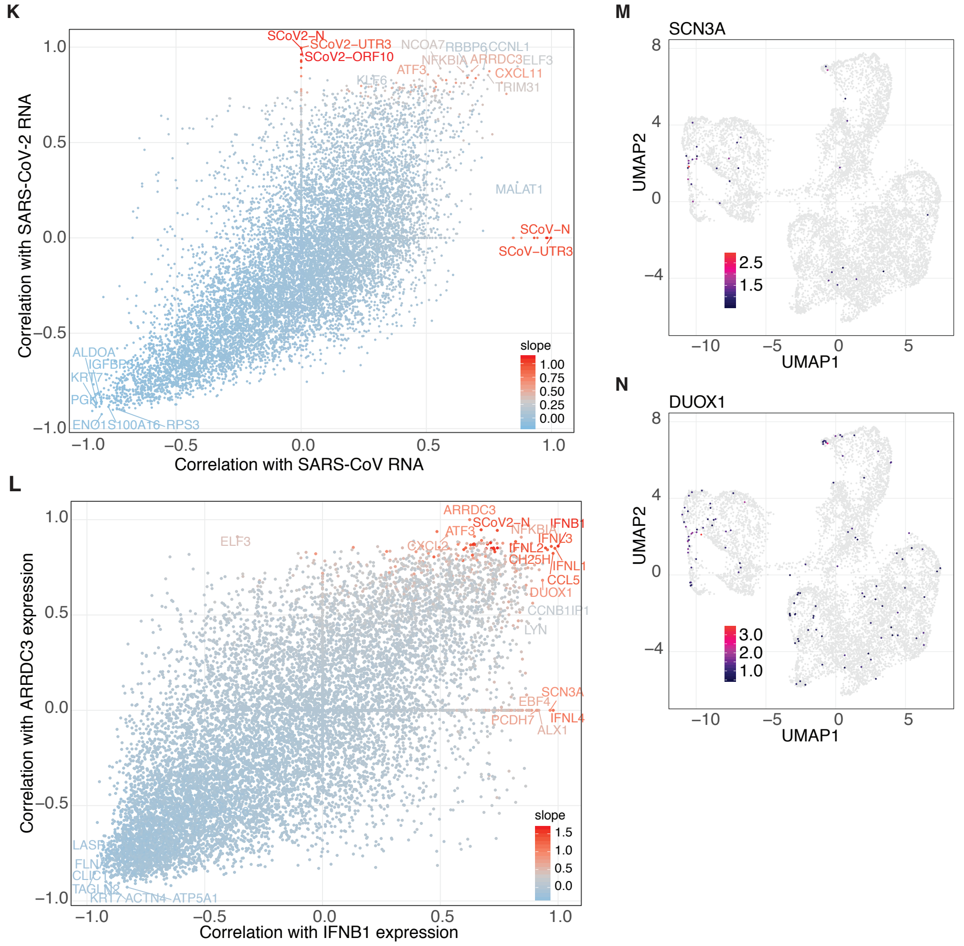

Figure S4. Single-cell RNA-sequencing shows different expression dynamics of genes induced by infection, Related to Figure 4

A, Percentages of cells bearing viral transcripts.

B, Relative densities of the percentage of viral transcripts per cell (log10 transformed) for SARS-CoV (left) and SARS-CoV-2 infected cells (right).

C, Projection as in Figure 4, with cells colored by replicate (upper panel) and type of infection (lower panel).

D, Cells from infected samples were split into groups with the amount of viral RNA either above (petrol) or below (red) the median. RNA molecule counts (UMI, unique molecular identifiers) were then displayed per group as boxplots. Median is shown as thick black line, lower and upper hinges correspond to the first and third quartiles, and whiskers to 1.5 times the inter-quartile range.

E-G, Projection as in Figure 4, but colored by expression values of the indicated genes.

K, Correlation of gene expression values with the amount of SARS-CoV RNA (x-axis) and SARS-CoV-2 RNA (y-axis).

L, Correlation of gene expression values with the amount of IFNB1 (x-axis) and ARRDC3 (y-axis).

M, N, Projection as in Figure 4, but colored by expression values of the indicated genes.

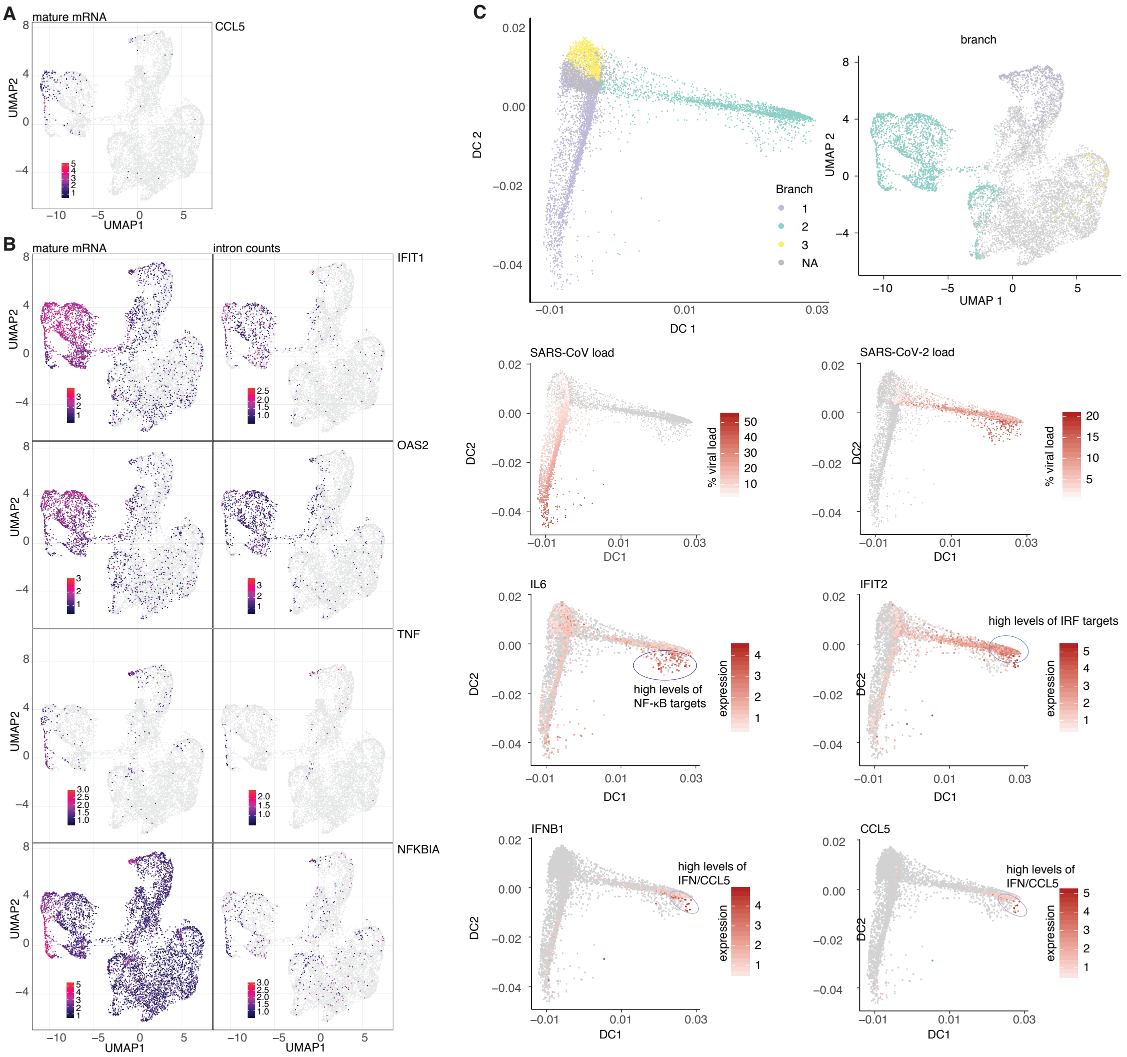

Figure S5. Interferon genes and NF-kappaB target genes are expressed in small subsets of cells, Related to Figure 5

AB, Projection as in Fig. 4, colored by the indicated mature mRNA expression values or intron counts for the indicated genes.

C, Branching analysis using destiny / diffusion maps. Except for the panel top right in C (projection as in Fig. 4 and 5), projections were done using diffusion components 1 (horizontal axis) and 2 (vertical axis). Areas of interest are marked by colored ellipses and labeled.

D, Velocity-based trajectories plotted on an embedding initialized with diffusion pseudo-time. Top panel: arrows represent trajectories based on RNA velocity, signal strength is represented by the length of the arrows as in Fig. 5A. Cells are colored by treatment. Other panels: cells are colored by expression levels of either viral RNA or host cell genes as indicated.

E, Projection using only host cell genes. Colored by mature mRNA expression values of the indicated genes.

F, Projection as in Fig. 4, colored my ACE2 mRNA (left) and intron signal (right).

G, Barplot showing fraction of cells containing intron reads from the ACE2 gene.

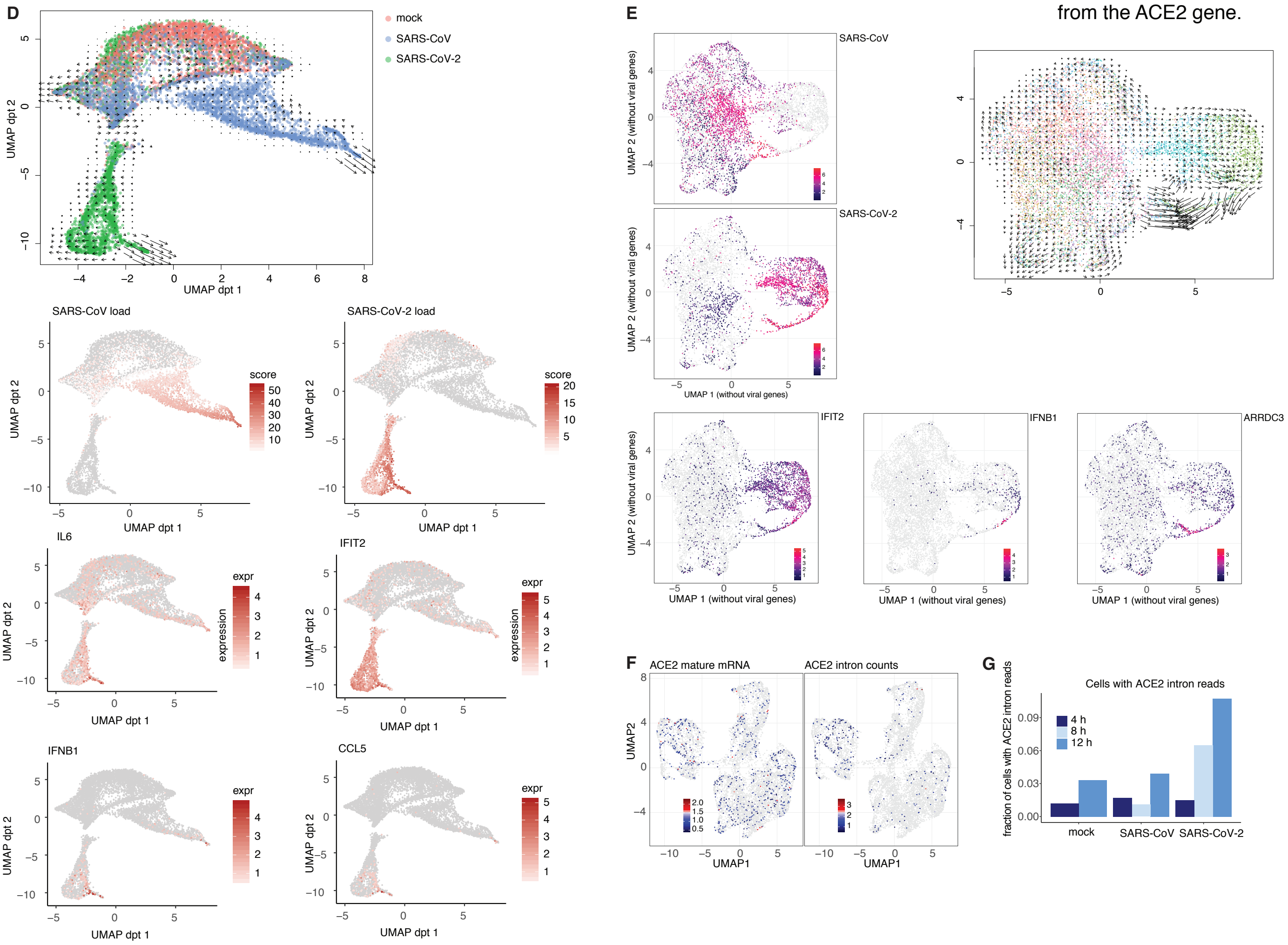

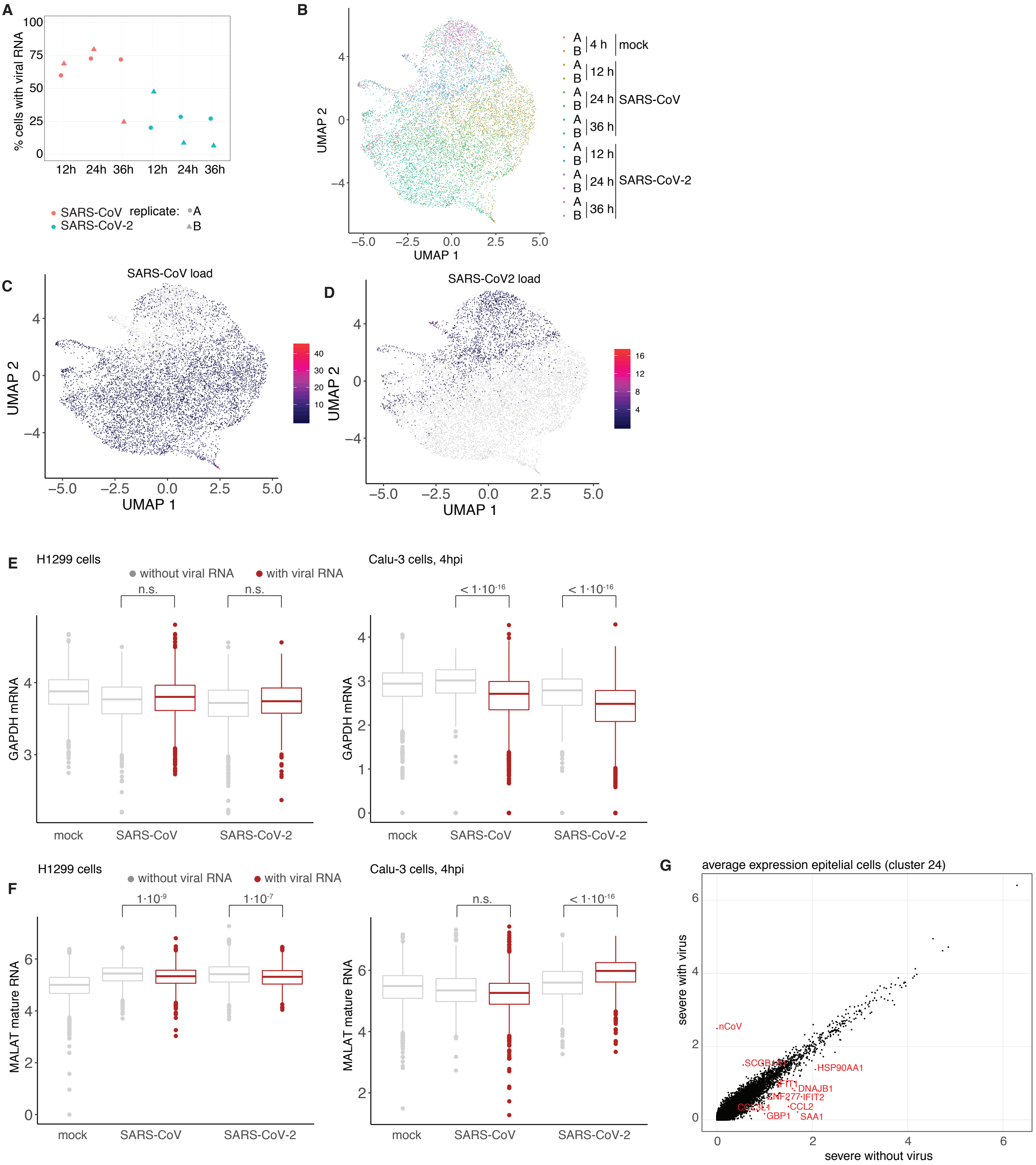

Figure S6. HSP90AA1 is deregulated in SARS-CoV-2 infected cells, Related to Figure 6

A, Percentages of cells with virus in the H1299 scRNA-seq data.

B-D, H1299 cells are projected in two dimensions using Uniform Manifold Approximation and Projection (UMAP) and colored as indicated.

EF, as in Fig. 6B and D, but for GAPDH and MALAT1 transcripts.

G, average gene expression values in epithelial cells/cluster 24 (see Supplementary Figure 2) of cells with and without viral reads. Expression of viral reads is represented by “nCoV”.

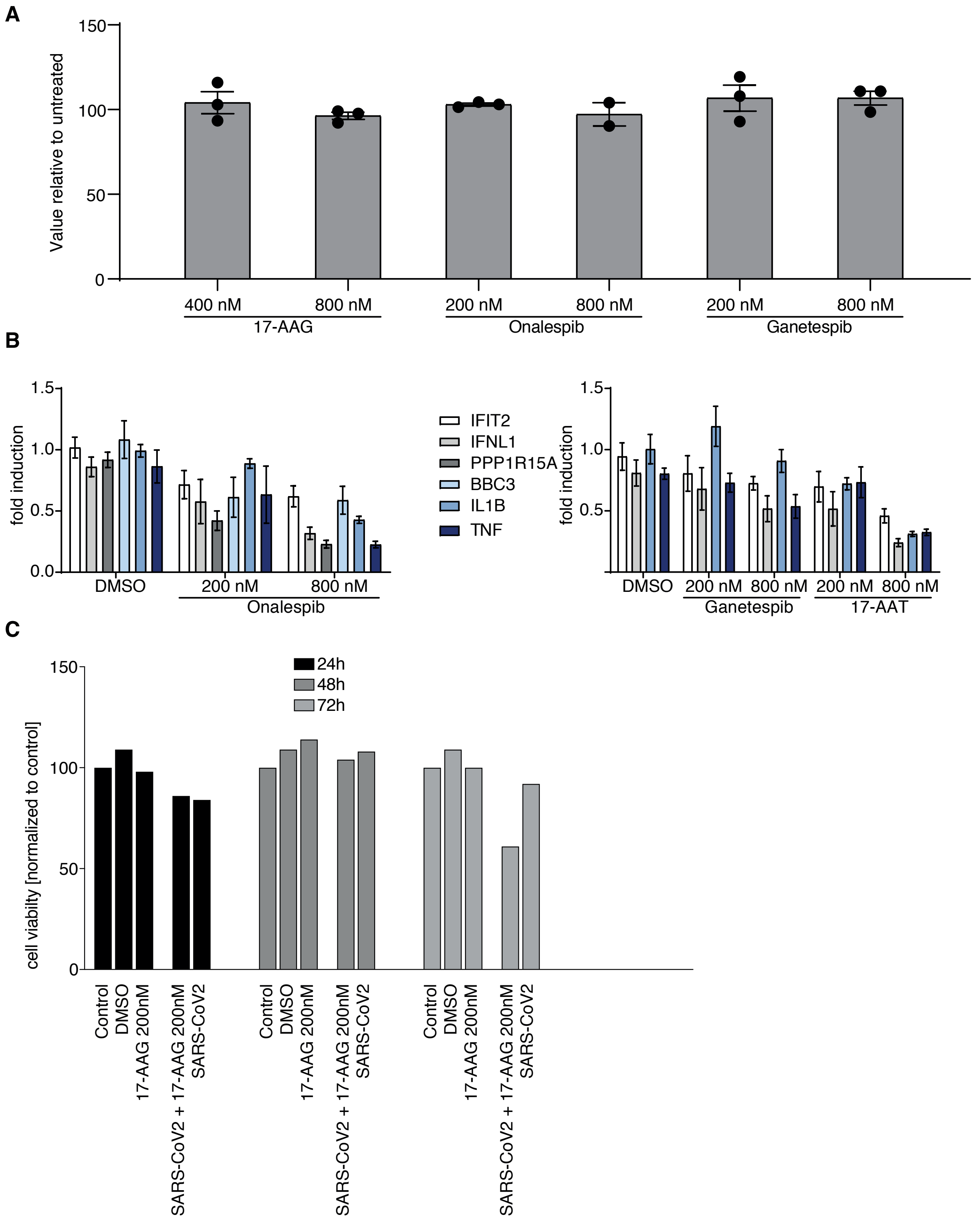

Figure S7. HSP90 inhibitors treatment reduce SARS-CoV-2 replication and induction of pro-inflammatory cytokines, Related to Figure 7
A, Cytotoxicity assay (CellTiter-Glo) of cells treated with the indicated HSP90 inhibitors for 16 hours.
B, Expression levels of selected mRNAs from the samples shown in Fig. 7 probed by RT-qPCR.
C, Cytotoxicity assay (CellTiter-Glo) of AECs treated with indicated treatments and time points.

Table S1, Related to Figure 1

Overview of datasets

Within file names, S1 means SARS-CoV-1 infection, S2 means SARS-CoV-2 infection, mock means mock infection (Vero cell supernatant), untr means untreated, i.e. no treatment at all

polyA RNA-seq datasets

Caco-2 cells

time points	conditions
4h	untreated, mock, SARS-CoV-1, SARS-CoV-2
12h	SARS-CoV-1, SARS-CoV-2
24h	mock, SARS-CoV-1, SARS-CoV-2

H1299 cells

Note: these samples were harvested in parallel with the scRNA-seq and proteomics

time points	conditions
4h	untreated, mock, SARS-CoV-1, SARS-CoV-2
12h	SARS-CoV-1, SARS-CoV-2
24h	SARS-CoV-1, SARS-CoV-2
36h	mock, SARS-CoV-1, SARS-CoV-2

Calu-3 cells series 1

Note: the same RNA was used to prepare the totalRNA and smallRNA data mentioned below. Cells that were detached after 24h were collected from the supernatant by centrifugation and processed separately.

time points	conditions
4h	untreated, mock, SARS-CoV-1, SARS-CoV-2
12h	SARS-CoV-1, SARS-CoV-2
24h	mock, SARS-CoV-1, SARS-CoV-2
24h	detached cells from SARS-CoV-1 and SARS-CoV-2

Calu-3 cells series 2

Note: the same RNA was used to prepare the totalRNA and smallRNA data mentioned below. Cells that were detached after 24h were collected from the supernatant by centrifugation and processed separately.

time points	conditions
4h	mock, SARS-CoV-1, SARS-CoV-2
8h	SARS-CoV-1, SARS-CoV-2
12h	mock, SARS-CoV-1, SARS-CoV-2

Total RNA-seq dataset (rRNA depletion)

Calu-3

Note: the same RNA was used to prepare the polyA RNA series 1 and smallRNA data

time points	conditions
4h	untreated, mock, SARS-CoV-1, SARS-CoV-2
12h	SARS-CoV-1, SARS-CoV-2
24h	mock, SARS-CoV-1, SARS-CoV-2

SmallRNA-seq dataset

Calu-3

Note: the same RNA was used to prepare the polyA series 1 and smallRNA data

time points	conditions
4h	untreated, mock, SARS-CoV-1, SARS-CoV-2
12h	SARS-CoV-1, SARS-CoV-2
24h	mock, SARS-CoV-1, SARS-CoV-2

Single-cell RNA-seq datasets

H1299 cells

Note: these samples were harvested in parallel with the polyA RNA-seq and proteomics. Count table is provided for cells with more than 2000 detected genes.

time points	conditions
4h	mock
12h	SARS-CoV-1, SARS-CoV-2
24h	SARS-CoV-1, SARS-CoV-2
36h	mock, SARS-CoV-1, SARS-CoV-2

Calu-3 cells

Note: Count table is provided for cells with more than 1000 detected genes, however the analysis in the manuscript was done only with cells with more than 2000 detected genes.

time points	conditions
4h	mock, SARS-CoV-1, SARS-CoV-2
8h	SARS-CoV-1, SARS-CoV-2
12h	mock, SARS-CoV-1, SARS-CoV-2

Proteomics

H1299 cells

Note: these samples were harvested in parallel with the scRNA-seq and polyA RNA-seq

time points	conditions
4h	mock
12h	SARS-CoV-1, SARS-CoV-2
24h	SARS-CoV-1, SARS-CoV-2
36h	mock, SARS-CoV-1, SARS-CoV-2

Table S2, Related to Figure 1
Percentage of virals read in the data presented here and a previously published dataset (GEO identifier GSE147507, only human samples)

polyA RNA-seq datasets

**Caco-2 cells**	% virus	**H1299 cells**	% virus	**Calu-3 cells series 1**	% virus	**Calu-3 cells series 2**	% virus
SARSCoV1-12h-A	26.53%	SARSCoV1-12h-A	0.09%	SARSCoV1-12h-A	35.79%	12h-SARSCoV1-1	5.00%
SARSCoV1-12h-B	27.18%	SARSCoV1-12h-B	0.12%	SARSCoV1-12h-B	38.17%	12h-SARSCoV1-2	5.40%
SARSCoV1-24h-A	35.67%	SARSCoV1-24h-A	0.13%	SARSCoV1-24h-A	48.13%	4h-SARSCoV1-1	0.33%
SARSCoV1-24h-B	34.60%	SARSCoV1-24h-B	0.14%	SARSCoV1-24h-A-sup	44.98%	4h-SARSCoV1-2	0.96%
SARSCoV1-4h-A	0.16%	SARSCoV1-36h-A	0.13%	SARSCoV1-24h-B	47.72%	8h-SARSCoV1-1	2.25%
SARSCoV1-4h-B	0.18%	SARSCoV1-36h-B	0.15%	SARSCoV1-24h-B-sup	51.72%	8h-SARSCoV1-2	3.10%
		SARSCoV1-4h-A	0.02%	SARSCoV1-4h-A	0.59%		
		SARSCoV1-4h-B	0.02%	SARSCoV1-4h-B	0.72%		
SARSCoV2-12h-A	10.96%	SARSCoV2-12h-A	0.06%	SARSCoV2-12h-A	32.89%	12h-SARSCoV2-1	35.77%
SARSCoV2-12h-B	12.80%	SARSCoV2-12h-B	0.05%	SARSCoV2-12h-B	32.97%	12h-SARSCoV2-2	39.63%
SARSCoV2-24h-A	22.39%	SARSCoV2-24h-A	0.04%	SARSCoV2-24h-A	16.48%	4h-SARSCoV2-1	1.58%
SARSCoV2-24h-B	22.69%	SARSCoV2-24h-B	0.04%	SARSCoV2-24h-A-sup	25.02%	4h-SARSCoV2-2	1.62%
SARSCoV2-4h-A	0.08%	SARSCoV2-36h-A	0.04%	SARSCoV2-24h-B	12.69%	8h-SARSCoV2-1	16.12%
SARSCoV2-4h-B	0.10%	SARSCoV2-36h-B	0.04%	SARSCoV2-24h-B-sup	16.18%	8h-SARSCoV2-2	15.16%
		SARSCoV2-4h-A	0.01%	SARSCoV2-4h-A	1.75%		
		SARSCoV2-4h-B	0.01%	SARSCoV2-4h-B	2.02%		

Total RNA-seq dataset (rRNA depletion)

Calu-3

Note: the same RNA was used to prepare the polyA RNA series 1 and smallRNA data

	% virus
SARSCoV1-12h-A	56.81%
SARSCoV1-12h-B	58.68%
SARSCoV1-24h-A	76.72%
SARSCoV1-24h-B	77.10%
SARSCoV1-4h-A	1.13%
SARSCoV1-4h-B	1.95%
SARSCoV2-12h-A	54.23%
SARSCoV2-12h-B	54.30%
SARSCoV2-24h-A	48.18%
SARSCoV2-24h-B	42.87%
SARSCoV2-4h-A	2.55%
SARSCoV2-4h-B	3.01%

GSE147507 samples MOI of 0.2 for 24 h

**Sample**	% virus
Series15_COVID19Lung_1	0.00006%
Series15_COVID19Lung_2	0.002%
Series1_NHBE_SARS-CoV-2_1	0.10%
Series1_NHBE_SARS-CoV-2_2	0.08%
Series1_NHBE_SARS-CoV-2_3	0.10%
Series2_A549_SARS-CoV-2_1	0.03%
Series2_A549_SARS-CoV-2_2	0.03%
Series2_A549_SARS-CoV-2_3	0.03%
Series5_A549_SARS-CoV-2_1	0.08%
Series5_A549_SARS-CoV-2_2	0.08%
Series5_A549_SARS-CoV-2_3	0.11%
Series6_A549-ACE2_SARS-Cov	53.60%
Series6_A549-ACE2_SARS-Cov	49.83%
Series6_A549-ACE2_SARS-Cov	57.49%
Series7_Calu3_SARS-CoV-2_1	13.19%
Series7_Calu3_SARS-CoV-2_2	17.01%
Series7_Calu3_SARS-CoV-2_3	14.58%

Table S3, Related to Figure 1

(provided as Excel Table)

Table S4, Related to Figure 4

Statistics scRNA-seq

H1299

						(more than 1 raw viral read count)		
						no virus	with S1	with S2
H1299-mock-4h-A	696	695	1	0	H1299-mock	99,86%	0,14%	0,00%
H1299-mock-4h-B	647	645	2	0	H1299-mock	99,69%	0,31%	0,00%
H1299-mock-36h-A	1924	1924	0	0	H1299-mock	100,00%	0,00%	0,00%
H1299-mock-36h-B	1081	1081	0	0	H1299-mock	100,00%	0,00%	0,00%
H1299-S1-12h-A	2354	944	1410	0	H1299-S1-12	40,10%	59,90%	0,00%
H1299-S1-12h-B	1665	515	1150	0	H1299-S1-12	30,93%	69,07%	0,00%
H1299-S1-24h-A	1050	287	763	0	H1299-S1-24	27,33%	72,67%	0,00%
H1299-S1-24h-B	1787	359	1428	0	H1299-S1-24	20,09%	79,91%	0,00%
H1299-S1-36h-A	3005	843	2162	0	H1299-S1-36	28,05%	71,95%	0,00%
H1299-S1-36h-B	793	596	197	0	H1299-S1-36	75,16%	24,84%	0,00%
H1299-S2-12h-A	1985	1586	0	399	H1299-S2-12	79,90%	0,00%	20,10%
H1299-S2-12h-B	1710	894	0	816	H1299-S2-12	52,28%	0,00%	47,72%
H1299-S2-24h-A	1363	976	0	387	H1299-S2-24	71,61%	0,00%	28,39%
H1299-S2-24h-B	1227	1118	0	109	H1299-S2-24	91,12%	0,00%	8,88%
H1299-S2-36h-A	2162	1577	0	585	H1299-S2-36	72,94%	0,00%	27,06%
H1299-S2-36h-B	1305	1217	0	88	H1299-S2-36	93,26%	0,00%	6,74%

Calu-3

						(more than 3 raw viral read counts)		
						no virus	with S1	with S2
Calu3-mock-4h-A	1454	1451	3	0	Calu3-mock-	99,79%	0,21%	0,00%
Calu3-mock-4h-B	839	838	0	1	Calu3-mock-	99,88%	0,00%	0,12%
Calu3-mock-12h-A	1074	1074	0	0	Calu3-mock-	100,00%	0,00%	0,00%
Calu3-mock-12h-B	732	728	0	4	Calu3-mock-	99,45%	0,00%	0,55%
Calu3-S1-4h-A	642	226	416	0	Calu3-S1-4h-	35,20%	64,80%	0,00%
Calu3-S1-4h-B	654	251	403	0	Calu3-S1-4h-	38,38%	61,62%	0,00%
Calu3-S1-8h-A	1428	0	1428	0	Calu3-S1-8h-	0,00%	100,00%	0,00%
Calu3-S1-8h-B	970	0	970	0	Calu3-S1-8h-	0,00%	100,00%	0,00%
Calu3-S1-12h-A	715	0	715	0	Calu3-S1-12h	0,00%	100,00%	0,00%
Calu3-S1-12h-B	807	0	807	0	Calu3-S1-12h	0,00%	100,00%	0,00%
Calu3-S2-4h-A	551	355	0	196	Calu3-S2-4h-	64,43%	0,00%	35,57%
Calu3-S2-4h-B	1170	736	0	434	Calu3-S2-4h-	62,91%	0,00%	37,09%
Calu3-S2-8h-A	948	0	0	948	Calu3-S2-8h-	0,00%	0,00%	100,00%
Calu3-S2-8h-B	510	0	0	510	Calu3-S2-8h-	0,00%	0,00%	100,00%
Calu3-S2-12h-A	767	0	0	767	Calu3-S2-12h	0,00%	0,00%	100,00%
Calu3-S2-12h-B	732	0	0	732	Calu3-S2-12h	0,00%	0,00%	100,00%

Transparent Methods

Cell culture

Vero E6 (ATCC CRL-1586), Calu-3 (ATCC HTB-55), Caco-2 (ATCC HTB-37) and H1299 (ATCC CRL-5803) were cultivated in Dulbecco's modified Eagle's medium (DMEM) supplemented with 10% heat-inactivated fetal calf serum, 1% non-essential amino acids, 1% L-glutamine and 1% sodium pyruvate (all Thermo Fisher Scientific) in a 5% CO_2 atmosphere at 37 °C.

Poly-I:C transfections

Transient transfection of eukaryotic cells was performed using X-tremeGENE™ siRNA transfection reagent (Roche) according to the manufacturer´s instructions. Briefly, 2x10^5 cells/ml were grown in 6-well plates for 24 h and fresh DMEM without antibiotics was added. OptiPRO SFM™ (Gibco) was supplemented with 0.25 µg poly(I:C) (Invivogen) and 0.75 µl X-tremeGENE™ siRNA reagent, incubated for 15 min, and 100 µl transfection mix was added to the cells.

RT-qPCR on intracellular RNA

RNA was isolated from Trizol using the RNA clean and concentrator kit (Zymo). The RNA was reverse transcribed using maxima RT and subjected to qPCR as described (Wyler et al., 2019). Primers used for qPCR are listed in supplementary table 5.

Viruses

SARS-CoV (Frankfurt strain, NCBI accession number AY310120) and SARS-CoV-2 (Patient isolate, BetaCoV/Munich/BavPat1/2020|EPI_ISL_406862) were used. For virus stock production, virus was grown on Vero E6 cells and concentrated using Vivaspin® 20 concentrators (Sartorius Stedim Biotech). Virus stocks were stored at -80°C, diluted in OptiPro serum-free medium supplemented with 0.5% gelatine (Sigma Aldrich) and phosphate-bufferd saline (PBS, Thermo Fisher Scientific). Titer was defined by plaque titration assay. Cells inoculated with cell culture supernatants from uninfected Vero cells mixed with OptiPro serum-free medium supplemented with 0.5% gelatine and PBS, in accordance to virus stock preparation, serves as mock infected controls. All infection experiments were carried out under biosafety level three conditions with enhanced respiratory personal protection equipment.

Virus growth kinetics and plaque titration assay

24 h prior to infection, the different cell lines were seeded to 70% confluence. The cells were washed once with PBS before virus (diluted in OptiPro serum-free medium) adsorption. After

incubation for 1 h at 37 °C, 5% CO2 the virus-containing supernatant was discarded and cells were washed twice with PBS and supplied with DMEM as described above.

To determine the amount of infectious virus particles in the supernatant a plaque titration assay was performed. For the assay Vero E6 cells were seeded to confluence and infected with serial dilution of virus-containing cell culture supernatant diluted in OptiPro serum-free medium. One hour after adsorption, supernatants were removed and cells overlaid with 2.4% Avicel (FMC BioPolymers) mixed 1:1 in 2xDMEM. Three days post-infection the overlay was removed, and cells were fixed in 6% formaldehyde and stained with a 0.2% crystal violet, 2% ethanol and 10% formaldehyde.

Western Blot Analysis

The expression of human ACE-2 (hACE-2) was confirmed by Western blot analysis. For the preparation of total cell lysate cells were washed with PBS and lysed in RIPA Lysis Buffer (Thermo Fisher Scientific) supplied with 1% Protease Inhibitor Cocktail Set III (Merck Chemicals). After an incubation of 30 min at 4 °C, cell debris were pelleted (10 min, 13,000 x g, 4 °C) and the supernatant transferred to a fresh reaction tube. For determining protein concentration Thermo Scientific's Pierce™ BCA Protein Assay Kit, according to the manufacturer's instructions was used. The protein lysates were mixed with 4xNuPAGE LDS Sample Buffer (Invitrogen) supplemented with 10% 2-mercaptoethanol (Roth). Protein lysates were separated by size on a 12% sodium dodecyl sulfatepolyacrylamid (SDS) gel and blotted onto a 0.2 µm polyvinylidene difluoride (PVDF) membrane (Thermo Scientific) by semi-dry blotting (BioRad). Primary detection of hACE-2 was done using a goat anti-hACE-2 antibody (1:1,250; #AF933, R&D Systems), a horseradish peroxidase (HRP)-labeled donkey anti-goat antibody (1:5,000, Dianova) and Super Signal West Femto Chemiluminescence Substrate (Thermo Fisher Scientific). As loading control, samples were analyzed for β-actin expression using a mouse anti-β-actin antibody (1:5,000, Sigma Aldrich) and a HRP-labeled goat anti-mouse antibody (1:10,000, Sigma-Aldrich).

Infections for RNA sequencing experiments

Calu-3 cells and H1299 cells were seeded at a concentration of 6 x 10^5 cells/mL and 5 x 10^4 cells/mL, respectively. 24 h post seeding cells were infected with SARS-CoV and SARS-CoV-2 at an MOI of 0.33 or Vero cell supernatant mixed with OptiPro serum-free medium supplemented with 0.5 % gelatine and PBS as negative control. 4, 8, 12 and 24 hpi samples were taken. For sequencing of total RNA the supernatant was removed and Trizol LS Reagent (Thermo Fisher Scientific) was applied to the cell-layer and incubated for 1 min at room temperature until the cells were lysed. The suspension was then transferred to a RNase

free reaction tube (Thermo Fisher Scientific) and stored at -80 °C. For scRNA-seq sample preparation the cells were washed with pre-warmed PBS, detached with pre-warmed trypsin for 3 min at 37 °C. The detached cells were transferred into a reaction tube (Eppendorf) and the following steps were performed on ice. Cells were spinned down at 1000 x g for 2 min at 4 °C, resuspended in PBS properly and passed through a 35 µm blue snap cap cell strainer (STEMCELL) and again pelletized The cell pellet was then resuspended in pre-chilled methanol (Roth) and stored at -80 °C.

RNA sequencing

Poly-A RNA sequencing

Poly-A RNA sequencing libraries were prepared using the NEBNext Ultra II Directional RNA Library Prep Kit (NEB) according to the manufacturer's protocols. Libraries were sequenced on a NextSeq 500 device at 1x76 cycles.

Small RNA sequencing

100 ng of total RNA of each condition was used for small RNA library preparation. Library preparation was performed using the SMARTer smRNA-Seq kit for Illumina from Clontech according to manufacturer's instruction. The small RNA libraries were pooled together with 19 % PhiX and sequenced on the NextSeq 500, 1 x 50 cycles.

Total RNA sequencing

1 µg of total RNA of each condition was used for total RNA library preparation. First, samples were depleted of ribosomal RNA using the RiboCop rRNA Depletion Kit (Lexogen) according to manufacturer's instruction. Following, ribo-depleted samples were processed with the TruSeq mRNA stranded kit from Illumina according to manufacturer's instruction. The total RNA libraries were sequenced on the HiSeq 4000, 2 x 76 cycles.

Viral RNA-seq analysis

Total and poly(A)$^{+}$ RNA-seq reads were mapped with STAR 2.7.3a to a combined genome comprised of GRCh38 and GenBank MN908947 (SARS-CoV-2) or AY310120 (SARS-CoV) using permissive parameters for noncanonical splicing [36, 101]. Viral genes were quantified by taking the top eight noncanonical splice events called by STAR across all total RNA-seq datasets according to the numbers of uniquely-mapping reads spanning the junction (Supplemental Table 3; note that for host cell gene expression analysis, also non-uniquely mapped reads were used). To estimate levels of ORF1ab, insertions, soft-clipping events and split reads were filtered from virus-mapping reads, followed by intersection with positions 53-83 of the virus using bedtools, requiring a minimum of 24 nucleotides overlap to reflect the parameters STAR requires to call a noncanonical splice junction [102]. These counts were either

combined with a count matrix of the human genes quantified by STAR and TMM/CPM normalized with edgeR (Figure S1D) or normalized by the total number of viral junction-spanning reads per time point (Figure S1E) [103]. Coverage plots were made from merged STAR-mapped BAM files, or from Bowtie-mapped small RNA-seq BAM files using ggsashimi [104]. This workflow was implemented with custom Python scripts in a Snakemake pipeline [105].

microRNA analysis

Raw reads were preprocessed by trimming with cutadapt (version 2.9) in two passes, first trimming i) the Illumina TruSeq adaptor at the 3' end and allowing for one mismatch,
ii) all 3'end bases with mean Phred score below 30 and iii) the three 5'end overhang nucleotides associated with the template-switching Clontech library preparation protocol.
In the second pass, poly(A)-tails were trimmed. Trimmed reads were mapped using bowtie (version 1.2.2) to a SARS genome consisting of the combined SARS-CoV and
SARS-CoV-2 genomes using the non-standard parameters (-q -n 1 -e 80 -l 18 -a -m 5 –best –strata). Reads that did not align to the SARS-CoV genome were aligned to the
GRCh38 genome. The expression of known miRNAs (miRBase 22 annotation) was estimated using mirdeep2 (version 2.0.0.7) and standard parameters.
The differential expression analysis used the limma [106] and edgeR [103] packages after applying the voom transformation to the TMM-normalized count data produced by mirdeep2.
For the different viral infections we contrasted SARS-CoV-2 24 h / SARS-CoV-2 4 h with mock 24 h / mock-4 h in order to test for those miRNAs differentially expressed long after the infection having removed any effects seen in mock as well.

TaqMan assays

TaqMan probes were purchased from ThermoFisher. 10 ng to 50 ng of total RNA were used for TaqMan assays and assays were performed according manufacturer's instruction with minor modifications. The minor modifications were: 10 mM dNTPs, Superscript III, 5 x FS buffer and Ribolock were used for the reverse transcription (RT) reaction and for 50 ng RNA input only 2 µl of the RT primer was used. All biological samples were handled in triplicates and the Ct values were normalized to the let-7a control.

Northern Blot

2,5 µg to 5 µg of total RNA were mixed equally with 2 x RNA loading dye, following denaturation for 5 min at 95 °C. The RNA of the denaturated samples was separated on 15 % urea polyacrylamide gels, transferred onto Hybond-N+ nylon membranes, UV crosslinked at 120.000 µJ/cm2 and probed with double digoxigenin (DIG)-labeled locked nucleic acid (LNA) detection probes (Qiagen, see supplementary table 5) at 55 °C over-night. The membranes were

subjected to stringent washes using SSC/SDS buffers. Subsequently, membranes were incubated with an anti-DIG-alkaline phosphatase (AP) solution (1:2500 diluted in blocking solution (Roche)) for 30 min at room temperature. Finally, northern blot signals were visualized using CDP star reagent (Roche) and the Vilber Fusion FX system according to manufacturer's instructions. The northern blot signals were normalized to the band intensity of the let-7a loading control.

Bulk RNA-sequencing analysis using DESeq2

Starting from count tables, RNA sequencing results were analysed on a per run basis comparing infected samples to time matched mock experiments unless otherwise specified using DESeq2 [107] version 1.22.2. Genes with a maximum read count across samples of less than two were filtered out. Differentially expressed genes were defined as genes with an absolute fold change in mRNA abundance greater than 1.5 (log_2 fold change of 0.58 - using DESeq2 shrunken log_2 fold changes) and an adjusted p-value of less than 0.05 (Benjamini-Hochberg corrected).

Gene ontology and KEGG enrichment analysis.

Genes whose mRNAs were found to be differentially expressed were subjected to gene set overrepresentation analysis using the clusterProfiler package in R [108].

Single-cell RNA-seq

Methanol-fixed cells were centrifuged at 2,000 x g for 5 min, rehydrated in 1 mL rehydration buffer containing 0.01% PBS/BSA and 1:100 Superasein (Thermo Fisher), and resuspended in 400 µL rehydration buffer followed by passing through a 40 µm cell strainer. Encapsulation was done with the Nadia system (Dolomite biosystems) using the built-in standard procedure. For library preparation, we followed the version 1.8 of the manufacturer's protocol, which is based on the protocol established by [109], with adding a second-strand synthesis step [110].

For the encapsulation, 75,000 cells in 250 µL rehydration buffer were used, with 250 µL of lysis buffer (6% Ficoll PM-400, 0.2% Sarkosyl, 20 mM EDTA, 200 mM Tris pH 7.5, 50 mM DTT) and 3 mL oil (Biorad #1864006). After encapsulation, beads were recovered from the emulsion by washing with 2 x 30 mL 6 x saline sodium citrate buffer (diluted from Sigma #S6639) buffer in a 5 µm ÜberStrainer (pluriSelect). After another washing step in 1.5 mL 6 x SSC, cells were washed with 5 x reverse transcription buffer (250 mM Tris pH 8, 375 mM KCl, 15 mM MgCl2, 50 mM DTT) and resuspended in 200 µL RT mix (50 mM Tris pH 8, 75 mM MgCl2, 3 mM MgCl2, 10 mM DTT, 4% Ficoll PM-400, 1 mM each dNTPs, 2.5 µM Macosko TSO, 10 µl Maxima H- RT enzyme). Beads were incubated for 30 min at room temperature and 90 min at 42 °C (all incubation steps on a rotator). After washing once with TE/0.5% SDS and twice with TE/0.01% Tween, beads were incubated in 200 µL exonuclease

mix (10µl Exonuclease in 1xexonuclease buffer, NEB #M0293) for 45 min at 37 °C, again on a rotator. After washing with once with TE/0.5% SDS and twice TE/0.01% Tween, beads were incubated for 5 min in 0.1 M NaOH, washed with TE/0.01% Tween and TE, and incubated in 200 µl second strand mix (50 mM Tris pH 8, 75 mM MgCl2, 3 mM MgCl2, 10 mM DTT, 12% PEG 8000, 1 mM each dNTPs, 10 µM dN-SMRT oligo, 5 µl Klenow enzyme NEB #M0212) for 1 h at 37 °C. Beads were again washed in TE/0.01% Tween and stored overnight in TE/0.01% Tween, then washed in TE and twice in water, and per sample eight PCR reactions with 4,000 beads each in 50µl using 1µM SMART PCR primer (oligos in supplementary table 5) and the 2x Kapa HiFi Hotstart Ready mix (Roche #07958935001) were performed, with pre-incubation for 3 minutes at 95 °C, then 4 cycles 98 °C/20s, 65°C/45s, 72 °C/3min, then 9 cycles 98 °C/20s, 67°C/20s, 72 °C/3min, then post-incubation for 3 minutes at 72 °C. The eight PCR reaction were pooled in three clean-up reactions using Ampure XP beads. For each oft the three sub-samples, a Nextera XT v2 (Illumina) reaction was performed with 600 pg DNA. In a 20µl reaction, 10 µl tagment DNA buffer and 5 µl amplicon tagment mix were incubated for 5 minutes at 55 °C, and, after addition of 5 µl neutralization buffer for 5 minutes at room temperature. Afterwards, 15 µl PCR master mix were added, 200 nM New-P5-SMART PCR hybrid oligo, 200 nM index oligo in total 50 µl. The Nextera reactions were then again pooled, purified using Ampure XP beads, and sequenced on a NovaSeq 6000 deviced with 21+71 cycles using Read1CustomSeqB for read 1.

Single-cell data analysis

After trimming one nucleotide from the 3' end of read one, count tables were generated using the PiGx-scRNA-seq pipeline [111] version 1.1.4. In short, cells are separated from empty barcodes using the inflection method as implemented in the dropbead package [112]. The reads are then mapped to the combined human and viral genome using the STAR aligner [101], with the default parameters. The resulting spliced and unspliced digital expression matrices are converted into loom, Seurat and SingleCellExperiment formats. For read mapping to the viral genomes, we used for SARS-CoV, the Frankfurt strain genome (accession number AY310120) and for SARS-CoV-2 the original Wuhan sequence (accession number MN908947.3). For both viruses, a feature labelled "UTR3" was added between the last annotated gene and the 3'-end, which captured most of the reads. Since the genes were counted separately, the scRNA-seq data contains counts for all genes in the annotation. Preprocessing was done in R (version 3.6), using the Seurat package [84]. Cells with less than 2000 unspliced reads were filtered out of the analysis. Raw reads were then normalized, and scaled. Variable genes were defined using the variance

stabilizing method. Dimensionality reduction was performed using diffusion maps, as implemented in the destiny Bioconductor [64] package.

Diffusion components were used as the basis for UMAP embeddings [113]. Pseudotime inference was performed using the diffusion pseudotime trajectory [65]. A secondary UMAP embedding was constructed by using, as input, the diffusion pseudotime calculated probabilities of cell-cell transitions. The UMAP was embedded using the python package umap-learn. All processing was done using the default parameters. The processing was done separately both with and without including SARS-CoV and SARS-CoV-2 viral genes. Viral load was calculated as the sum of detected viral reads in each cell. Velocity estimation was performed using the R implementation of the original Velocyto method [62]. The following parameters were used for the projection of the vector field: n = 200, scale = 'sqrt', arrow.scale = 3, min.grid.cell.mass = 0.5.

To increase the power of detecting dynamic changes in gene expression (Fig. 5E), pseudotime based embedding was dynamically discretized using the Louvain algorithm. The input to the Louvain algorithm are eigenvectors of the diffusion matrix. The resulting bins were ordered based on the median SARS-CoV-2 expression, and the percentage of cells expressing genes of interest was visualized in each of the bins. The ordering of bins based on the median SARS-CoV-2 load corresponded almost exactly to the velocyto inferred trajectories.

Both Calu3 and H1299 cells were analyzed using the same parameters. To detect changes happening early in infection, Calu3 4h cells were additionally separately analyzed.

Preprocessing and analysis/figure scripts are available at github (https://github.com/BIMSBbioinfo/Ewyler_SARS-CoV).

The analysis was visualized using ggplot2 [114], ggrepel [115], and the interactive exploration was enabled by iSEE [116].

HSP90 inhibitor experiments

The HSP90 inhibitors were purchased either from Sigma (17-AAG, A8476) or from Selleckchem (Onalespib, S1163 and Ganetespib, S1159), and dissolved in DMSO. Cells were seeded and grown to subconfluence and infected with SARS-CoV-2 MOI 0.01 (Calu-3) or MOI 0.5 (AECs) diluted in OptiPro serum free medium. After 1 h virus adsorption the supernatant was removed and cells were washed twice with PBS. DMEM containing dilutions of 17-AAG (200 nM, 400 nM, 800 nM, 2,000 nM) or DMSO as solving control. Samples for detection of viral RNA and infectious particles in the supernatant as well as total RNA within the cells were taken 8, 16 and 24 hpi.

The cytotoxicity of the the HSP90 inhibitor was assured by cell viability assay using CellTiter-Glo® Luminescent Cell Viability Assay according to manufacturer's instruction (Promega). The activity of untreated cells was set as 100% and cells were treated with different concentrations of 17-AAG. The viability of cells was measured 16 and 24 h after treatment using Mithras Luminescence microplate reader (Berthold).

Primary airway epithelial cells

Cells isolated from distal lung tissue were cultured as described in Imai-Matsushima et al., 2018. Briefly, for expansion primary cells were co-cultivated with gamma-irradiated mitotically inactivated NIH3T3 mouse embryonic fibroblasts (MEFs) in a 3:1 mixture of Ham's F-12 nutrient mix (Life technologies) and DMEM supplemented with 5% FCS, 0.4µg/mL hydrocortisone (Sigma-Aldrich), 5µg/mL recombinant human insulin (Sigma-Aldrich), 8.4 ng/mL cholera toxin (Sigma-Aldrich), 24µg/mL Adenine (Sigma-Aldrich), and 10ng/mL recombinant human epidermal growth factor (Invitrogen), 0.1 µM DBZ (Tocris) and 9 µM Y27632 (Miltenyi Biotec)

Differentiation was induced by additional treatment with 3 µM CHIR-99021 (Sigma), 10 ng/ml KGF (Invitrogen), 10 ng/ml FGF-10 (Invitrogen), 100 µM IBMX (Sigma), 100 µM 8-Bromoadenosine 3',5'-cyclic monophosphate (Biolog), 25 nM Dexamethason (Sigma) and 20 µM DBZ for 3 days. Two days prior to infection the primary cells were separated from the MEFs by differential trypsinization and subsequently seeded in cell culture vessels in DMEM with 10% FCS, 1% non-essential amino acids, 1%, L-glutamine and 1% sodium pyruvate.

RT-qPCR of viral RNA in the supernatant

The viral RNA from supernatant of infected cells was isolated using the NucleoSpin RNA virus isolation kit (Macherey-Nagel) according to the manufacturer's instructions. To determine the amount of viral genome equivalents the previously published assay specific for both SARS-CoV and SARS-CoV-2 Envelope gene [117] was used. Data analysis was done using LightCycler Software 4.1 (Roche).
